# Positive Effects of Nature on Cognitive Performance Across Multiple Experiments: Test Order but Not Affect Modulates the Cognitive Effects

**DOI:** 10.3389/fpsyg.2019.01413

**Published:** 2019-07-03

**Authors:** Cecilia U. D. Stenfors, Stephen C. Van Hedger, Kathryn E. Schertz, Francisco A. C. Meyer, Karen E. L. Smith, Greg J. Norman, Stefan C. Bourrier, James T. Enns, Omid Kardan, John Jonides, Marc G. Berman

**Affiliations:** ^1^Department of Psychology, University of Chicago, Chicago, IL, United States; ^2^Department of Neurobiology, Care Science and Society, Aging Research Center, Karolinska Institute, Stockholm, Sweden; ^3^Department of Psychology, Stockholm University, Stockholm, Sweden; ^4^Department of Psychology, University of British Columbia, Vancouver, BC, Canada; ^5^Department of Psychology, University of Michigan, Ann Arbor, MI, United States

**Keywords:** cognitive restoration, cognitive performance, directed attention, nature, environment, affect, practice effects, order effects

## Abstract

Interactions with natural environments and nature-related stimuli have been found to be beneficial to cognitive performance, in particular on executive cognitive tasks with high demands on directed attention processes. However, results vary across different studies. The aim of the present paper was to evaluate the effects of nature vs. urban environments on cognitive performance across all of our published and new/unpublished studies testing the effects of different interactions with nature vs. urban/built control environments, on an executive-functioning test with high demands on directed attention—the backwards digit span (BDS) task. Specific aims in this study were to: (1) evaluate the effect of nature vs. urban environment interactions on BDS across different exposure types (e.g., real-world vs. artificial environments/stimuli); (2) disentangle the effects of testing order (i.e., effects caused by the order in which experimental conditions are administered) from the effects of the environment interactions, and (3) test the (mediating) role of affective changes on BDS performance. To this end, data from 13 experiments are presented, and pooled data-analyses are performed. Results from the pooled data-analyses (*N* = 528 participants) showed significant time-by-environment interactions with beneficial effects of nature compared to urban environments on BDS performance. There were also clear interactions with the order in which environment conditions were tested. Specifically, there were practice effects across environment conditions in first sessions. Importantly, after parceling out initial practice effects, the positive effects of nature compared to urban interactions on BDS performance were magnified. Changes in positive or negative affect did not mediate the beneficial effects of nature on BDS performance. These results are discussed in relation to the findings of other studies identified in the literature. Uncontrolled and confounding order effects (i.e., effects due to the order of experimental conditions, rather than the treatment conditions) may explain some of the inconsistent findings across studies in the literature on nature effects on cognitive performance. In all, these results highlight the robustness of the effects of natural environments on cognition, particularly when confounding order effects have been considered, and provide a more nuanced account of when a nature intervention will be most effective.

## Introduction

Interactions with nature have been found to be beneficial to different aspects of health and well-being across experimental and epidemiological studies, including improved mental health and affective states (Alcock et al., 2014; McMahan and Estes, 2015), reduced stress (Jiang et al., 2016), and better cardiovascular and metabolic health (Kardan et al., 2015; Shanahan et al., 2016; Crouse et al., 2017). Additionally, improvements have been observed for executive cognitive performance, including directed (also referred to as “executive”) attention (Berman et al., 2008, 2012; Bratman et al., 2012).

Attentional processes historically have been separated into two branches: involuntary attention, in which attention is captured by salient stimuli, and voluntary or directed attention, in which attention is directed by top-down executive cognitive control processes required for higher-order executive cognitive functions.

It has been argued that directed attention capacity, or “executive attentional control,” represents a common central construct measured by WM span tasks (Conway et al., 2005; McCabe et al., 2010; Gazzaley and Nobre, 2012) and that WM is a system for “attention to memory” rather than a memory system in itself (Oberauer et al., 2007; Souza and Oberauer, 2016). That is, directed attention contributes to the activation/building, maintenance, updating and retrieval of representations, and arbitrary bindings in WM (Oberauer et al., 2007; Jonides et al., 2008; Souza and Oberauer, 2016). For example, the Backwards Digit Span (BDS), which is considered a classic WM span task, is extremely taxing on directed attention as it requires encoding, maintenance, active processing to reverse the digit order, and updating of information in working memory. Additionally, WM span tasks are associated with performance on other tasks tapping directed attentional control (Conway et al., 2005; Chein et al., 2011).

Importantly, the capacity to control attention has been found to vary across time within individuals, and environmental exposure may be one cause of this variation. Specifically, attention restoration theory (ART) (Kaplan, 1995) has posited that interacting with natural environments can facilitate improvement in directed attention and restore directed attention from fatigue. It has been argued that natural environments have this restorative potential because they consist of inherently fascinating stimuli (e.g., trees, flowers, water) that modestly draw upon bottom-up involuntary attention, allowing directed-attention mechanisms a chance to replenish. In addition, many natural environments consist of few alerting stimuli that require directed attention to monitor behavior appropriately and therefore place less demands on directed-attention resources (e.g., in urban environments having to attend to traffic and other people to prevent collisions, etc.). Thus, following an interaction with natural environments, individuals may perform better on tasks that depend on directed-attention control processes, such as WM tasks. Urban environments, on the other hand, tend to contain bottom-up stimulation that captures attention dramatically and/or requires the processing of multiple sources of information (e.g., monitoring different human activities), which require directed attention to evaluate and filter out or act on different stimuli and stimulus-driven impulses (e.g., traffic, people, advertisements, etc.), thus making urban environments less restorative.

However, as noted above, the reported effects of natural environments on cognitive performance have varied across studies (see e.g., Bratman et al., 2012; Ohly et al., 2016; Stevenson et al., 2018). Variable findings may stem from differences in sample sizes/power, type of cognitive assessment, as well as study designs and procedures. Design elements that may influence the amount of restorative benefits observed, may be the amount of natural elements in the exposure, the duration of the exposure, and the exposure type (e.g., being in real natural settings, vs. being exposed to pictures, videos or sounds of nature, and/or other depictions of nature). For example, in a meta-analysis of the effects of natural environments on affect, McMahan and Estes (McMahan and Estes, 2015) concluded that exposure to real-world nature (i.e., being in a natural park) yields greater improvements in affect compared to artificial nature exposures (e.g., images). Furthermore, Jiang, et al. found that a higher dosage of natural stimuli (higher compared to lower levels of tree cover density) was more restorative in terms of lower perceived stress levels (Jiang et al., 2016).

When testing the effects of different experimental conditions on cognitive performance, different study designs and procedures may also introduce different sources of error variance in the dependent measure. While within-subjects repeated measures designs are more powerful, repeated testing of cognitive performance in the same individuals across one or multiple sessions tends to introduce variance in cognitive performance that can be attributed to practice effects, and not simply due to the experimental manipulation of the interleaving environment interaction. Such practice effects are rather well documented (see e.g., Lemay et al., 2004; Beglinger et al., 2005; Falleti et al., 2006; Bartels et al., 2010; Woods et al., 2011) and the greatest improvements in performance tend to occur from the first to the second test administration, that is, in the first testing session of an environment exposure condition (if participants had no prior practice in the specific tests employed). As such, order effects are introduced on the cognitive outcome measure, since the order of experimental conditions will determine during which condition the initial practice effects will occur for each participant. Order effects may also occur due to changes in participants' expectations from the first to the second experimental condition. Controlling for such order effects, however, requires greater sample sizes, which can be achieved when analyzing the data from multiple experiments with similar experimental designs and dependent measures.

In a recent review and meta-analysis of studies of attention restoration from nature vs. control environment exposures, which included a broad range of study designs and cognitive measures, significant evidence of cognitive improvement was demonstrated after nature compared to control conditions on the following cognitive measures: BDS, forward digit span (FDS) and trail making test B (TMTB) (Ohly et al., 2016). However, in the review by Ohly et al., analyses were done only for post-environment cognitive performance, thus not testing for differences in performance *changes* from before to after nature vs. control environment interactions. The authors also highlighted that the great heterogeneity in study designs and methods limited the meta-analysis and conclusions that could be drawn. For example, the meta-analysis of each type of cognitive measure was limited to only a few studies, due to the heterogeneity in cognitive tests used and the data reporting measures across studies (Ohly et al., 2016). Another recent meta-analysis found that tasks in the domains of working memory, cognitive flexibility, and attentional control were most likely to show improvements after exposure to natural vs. urban environments (Stevenson et al., 2018). However, in neither of these papers were analyses conducted in order to delineate the effects of test order from the effects of environment exposure. Additionally, these studies did not report data by test order, which prevents analyses of this factor.

In this paper, we aimed to further the knowledge of nature and urban environmental effects on executive cognitive performance. We did so by performing pooled data-analyses on all of our own labs' published and new/unpublished experimentally controlled studies testing the effects of natural vs. urban environments on directed attention performance using a consistent working memory (WM) task—the backwards digit span (BDS). We included studies in which: (1) the effects of natural environment exposures/interactions on directed attention were tested using the same cognitive test (BDS, which places high demands on directed attention), and (2) the designs were randomized controlled experiments with pre- and post-exposure testing and control conditions, allowing for comparability across studies and analyses of overall effect sizes. Analyzing these datasets together allows for addressing questions that may not be evaluated in single isolated studies, or studies with too much heterogeneity in designs and methods.

Interacting with natural environments has also been found in experimental studies to significantly improve affective state—by increasing positive affect, decreasing negative affect, or both (e.g., McMahan and Estes, 2015). Observational studies have also found that people are happier in greener environments (MacKerron and Mourato, 2013), and that park visits are associated with positive, reflective thinking (Schertz et al., 2018). Affective state, can in turn have an impact on cognitive performance, including directed attention (Brose et al., 2012, 2014). The role of affective changes is thus an important factor to consider when investigating changes in cognitive performance after different environmental exposures, and may be evaluated more robustly in pooled analyses of multiple studies, yielding greater statistical power. Thus, an additional purpose of this paper is to test the effects of environmental condition on positive and negative affect, and specifically if such effects can explain (and may mediate) the effects of the environmental conditions on cognitive performance. Such analyses provide greater knowledge of how natural environment interventions impact performance and by what mechanisms, which have theoretical and practical implications.

Findings from other studies assessing the effects of nature vs. control conditions on BDS performance and other objective measures of directed attention performance were also reviewed and integrated with the findings from the pooled data and analyses of the multiple experiments reported in this paper.

In summary, the specific purposes of this paper were to: (1) evaluate the effects of nature vs. urban interactions/exposures on directed attention performance (using the BDS task) across multiple experiments/studies, (2) evaluate and delineate potential effects of order/practice on cognitive performance from effects of the environment conditions/exposures, (3) evaluate the independence (or dependence) of the effects on cognitive performance (BDS) from the effects on affective state from nature vs. urban exposures, and (4) compare the effects between studies using different exposure types.

## Methods

### Study Samples

The studies presented in this paper include all studies/datasets collected by Berman et al. (published and new/unpublished), in which the effects of nature vs. urban environment exposures were tested on directed attention in controlled experimental settings (to ensure the validity of the cognitive performance measures). All studies employed a randomized crossover trial (RCrT) or a randomized controlled trial (RCT) design and employed the same cognitive test to evaluate effects on directed attention—the BDS task. The consistency in the methods across the presented studies enable pooled data analyses as well as direct comparison of cognitive performance scores/changes across different studies.

A summary of the studies and sample characteristics are provided in Table 1. For studies which have been published previously or which have been submitted for publication, the reference is included in the column of study names. For more details on the study samples that have been published previously, see the original research articles (Berman et al., 2008, 2012; Bourrier et al., 2018; Van Hedger et al., 2018).

**Table 1:** Study and sample characteristics.

**STUDY/Sample, place, year of data collection/publication**	**Valid N (after excl. Participants with incomplete BDS data and outliers)**	**Age**	**Gender**	**Env W or B Subj Factor**	**Env stimuli and duration**	**Env info**	**BDS admin (max test score)**	**Attrition/outliers**
1. Walk, UM. (Berman et al., 2008)	37 UM students	M = 22.62 y (before excl 1 outlier)	23 F, 15 M (before excl 1 outlier)	W	Walk, 50–55 min	Nature: Nichols Arboretum, Ann Arbor. Urban: Downtown Ann Arbor. Michigan, USA	AS, VerbR, IPT (14)	1 outlier
2. Picture study, UM. (Berman et al., 2008)	12 UM students	M = 24.25 y	8 F, 4 M	W	Pic, 10 min	Nature: scenery of Nova Scotia. Urban: pictures of Ann Arbor, Detroit, and Chicago.	AS, VerbR, IPT (14)	
3. Walk, healthy sample, UM, 2011.	21 UM students	M = 23.62 y, SD = 6.62	11 F, 10 M	W	Walk, 50–55 min	Nature: Nichols Arboretum, Ann Arbor. Urban: Downtown Ann Arbor. Michigan, USA	AS, VerbR, IPT (14)	
4. Walk, MDD sample, UM. (Berman et al., 2012)	19 diagnosed with MDD, from UM and greater Ann Arbor area.	M = 26 y (before excl of 1 participant)	12 (63.2%) F, 7 (36.8%) M	W	Walk, 50–55 min	Nature: Nichols Arboretum, Ann Arbor. Urban: Downtown Ann Arbor. Michigan, USA	AS, VerbR, IPT (14)	1 outlier
5. Picture study, UC, 2015.	45 UC undergrad.students	M = 19.84, SD = 1.07	29 F, 16 M	W	Pic, 8–10 min	50 Nature vs. 50 Urban/built environment pictures.	AS, KR, CT (14)	1 outlier
6. Picture study, UM, 2015.	37 UM undergrad.students	Missing	30 F, 7 M	W	Pic, 8–10 min	50 Nature vs. 50 Urban/built environment pictures.	AS, KR, CT (14)	1 outlier
7. Walk, UC, 2016.	49 UC undergrad students	18–26 y, M = 19.57, SD = 1.81	31 (63.3%) F, 18 (36.7%) M	W	Walk, 15–20 min	Nature: Univ. of Chicago main quadrangles (grass, trees, pond). Urban: walk along the streets outside of the Univ. Of Chicago main quadrangles.	AS, KR, CT (18)	6 excluded due to doing the wrong walk.
8. Virtual Reality (VR) study 1, UC, 2016.	82 UC undergrad students	18–24 y, M = 19.67, SD = 1.70	47 F, 32 M (3 missing)	W	VR, 10 min	Nature: VR path with surrounding trees, grass and water ponds. VR Urban: city block with streets and buildings.	AS, KR, CT (18)	1 outlier
9. Virtual Reality study 2- with habituation, UC, 2016.	82 UC undergrad students	18–28 y, M = 19.57 y, SD = 3.76 (missing age for 19 participants)	53 F, 29 M	B	VR, 10 min	Ibid. Session 1's = VR vs. no-VR habituation in control/space env., session 2's = Nature or Urban VR env.	AS, KR, CT (18)	1 outlier
10. Composite study- Sounds. UC. (Van Hedger et al., 2018)	44 UC undergrad students	18–44 y, M = 21.35, SD = 4.34	24 F, 17 M, 3 no answer	B	Sounds, 20–25 min	Nature: nature sounds, e.g., bird song. Urban: urban sounds, e.g., traffic)	AS or VS, KR, CT (14)	
11. Composite study- Pictures. UC, 2016.	40 UC undergrad students	18–31 y, M = 21.13, SD = 3.06	28 F, 12 M	B	Pic, 20–25 min	Nature: 100 pics of nature scenes. Urban: 100 pics of built environment scenes.	AS or VS, KR, CT (14)	
12. Video study, UBC. (Bourrier et al., 2018)	60 (+30 in a control condition) from UBC human subject pool, the Reservax subject pool and poster advertising.	17–45 y, M = 21.1, SD = 3.54	67 (74 %) F, 23 M	B	Video (no sound), 10 min	Nature: Banff National Park tour, Alberta Canada. Urban: Tour of Barcelona.	VS, KR, CT (14)	
13. Picture dose study- Session 1 and 2, UM 2009.	39 UM undergrad.students	Missing	Missing	Nature × 2 sessions	25–100 pics, 5–20 min	Short = 25 pics, 4–5 min; Medium = 50 pics, 8–10 min; Long = 100 pics, 16–20 min.	AS, VerbR, CT (14)	2 did not do 2nd session
**Total, env studied as a W factor** (study 1–8)	**302**							
**Total env studied as a B factor** (study 9–12, excl Pic dose study)	**226**							
**Grand Total**	**567 (+30)**							

*Env, Environment condition; W, within-participant factor; B, between-participant factor; UM, Univ. of Michigan; UC, Univ. of Chicago; MDD, major depressive disorder; IPT, in-person testing; CT, computerized test (using E-prime); AS, auditory stimuli; VerbR, verbal response; KR, keypress response*.

### Procedures in the Studies

All studies measured cognitive performance with the BDS, and affective state (except one study: Bourrier et al., 2018) with the PANAS, before and after controlled exposures to natural environments and non-natural/urban environments, with environment type (nature, urban/other) being a within- or a between-subjects factor depending on the study. Participants were randomly assigned to the experimental conditions, and the order of conditions was counterbalanced for within-participant designs. A summary of study characteristics, including the type and duration of environmental exposures and study design, are provided in Table 1.

For new and previously unpublished studies, additional descriptions of procedures and methods are provided in Appendix A in Supplementary Material. For more details on the methods in studies that have been published previously, please see the original research articles (Berman et al., 2008, 2012; Bourrier et al., 2018; Van Hedger et al., 2018).

### Measures

#### BDS

Participants in each study were tested on the backwards digit span task, in which participants are presented with number sequences which they were required to repeat in backwards order. Fourteen to eighteen (depending on study) separate number sequences (trials) were presented. Number sequences were a minimum of three digits in length and with a maximum of 9–11 digits (depending on the study), with two trials at each digit string length. The “all-or-nothing unit scoring” method (Conway et al., 2005) was used, in which a participant receives one point for each trial sequence correctly recalled, and no partial credit is given for recalling some portion of the digit string correctly (i.e., the entire digit string must be correct). This is a general performance measure that taps both working memory capacity and directed attention, since inconsistent performance is also reflected in this measure. See Table 1 for information on test administration in the respective studies. The BDS task was used in the studies because this task places high demands on executive attentional control processes (including encoding, maintenance, manipulation and updating of to-be-remembered items), and would therefore be sensitive to changes in this cognitive capacity caused by different environmental interactions. Furthermore, the task has been used consistently across the different studies in order to be able to pool as well as compare results across studies.

#### PANAS

The Positive Affect and Negative Affect Schedule, PANAS (Watson et al., 1988), was used to assess participants' affect in all except one sample (the video study, at the University of British Columbia by Bourrier et al., 2018, where no affect measures were recorded). Affect-related adjectives (e.g., enthusiastic) were rated on a scale of 1 to 5 for how well each adjective described participants' current affective state (1 = very slightly or not at all, 5 = extremely). There were 10 positive and 10 negative affect items.

### Statistical Analyses

For each respective study sample, only participants with complete data for the BDS were included in the analyses. Statistical analyses were computed using SPSS 24.

Data sets were screened for univariate (± 3.25 SD) and multivariate outliers (using Mahalanobis distance, evaluated using the Chi-squared distribution and a drop-out threshold of *p* < 0.001), and outliers were subsequently excluded in further statistical analyses (see Table 1 for outliers in each respective dataset).

Analysis of variance (ANOVA) models were computed (in the general linear models procedure in SPSS) to evaluate the effects of environmental condition (nature vs. urban) across time (from pre- to post environment exposure) on BDS performance and affective state across the different studies.

Data from studies with a randomized crossover design with environment as a repeated/within-subjects factor vs. randomized controlled studies with environment as a between-subjects factor were analyzed in separate models—modeling the environmental factor as a within-subjects vs. a between-subjects factor as appropriate, and the time point (pre- vs. post environment interaction) as a within-subjects factor.

For the study samples in which environment was a within-subjects factor, the order of environmental conditions was included as a between-subjects factor in an additional model to evaluate the main and interaction effects of the order of environmental condition (i.e., natural environment exposure in the first or second session).

The Linear Mixed effects Model (LMM) procedure in SPSS was utilized to analyze the same factorial models as described above, *while also adjusting for affective state at each measurement point*. Unlike repeated measures ANOVAs (which cannot handle time-varying covariates), in the LMM models we included affect (measured pre- and post each environment exposure) as a time-varying covariate in the factorial model. These models were computed in order to evaluate if the effects of environmental condition on cognitive performance were independent of or mediated by (i.e., co-varied with) changes in affective state. For studies with a randomized crossover design, the fixed factors time (pre- vs. post environment exposure) and environment (nature vs. urban) were specified as repeated-measures fixed factors (using unstructured covariance as the covariance structure, IBM, 2018), and the order factor (i.e., participating in the nature condition first vs. the urban condition first) was specified as a between-subjects factor. The full factorial models included the factors: time, environment, and order as the independent variables with BDS scores as the dependent variable and with affect scores included as time-varying covariates. These models were computed using maximum likelihood model estimation. For data analyses where environment was tested as a between-subjects factor, only time (pre- vs. post-environment interaction) was specified as a repeated measure. Further descriptions and syntax for these analyses are provided in Appendix A in Supplementary Material.

Since the picture dose-response study did not test the contrast between nature and urban conditions, and only tested the effects of nature picture viewing in 2 sessions, this sample was not included in the pooled data-analysis models. Results for this study sample are presented individually for comparison purposes only (see Tables 1, 2).

**Table 2:** Mean BDS scores by study, time (pre- vs. post environment exposure), environment condition, and order (test session).

				**Nature 1st/Urban 2nd**						**Urban 1st/Nature 2nd**						**Other control**		
				**Nature (session 1)**			**Urban (session 2)**			**Nature (session 2)**			**Urban (session 1)**			**Other (session 1)**		
**STUDY/Sample**	**Valid N**	**Env factor**	**Statistic**	**Pre**	**Post**	**Pre-post diff**	**Pre**	**Post**	**Pre-post diff**	**Pre**	**Post**	**Pre-post diff**	**Pre**	**Post**	**Mean Diff**	**Pre**	**Post**	**Pre-post diff**
1. Walk, UM. Berman et al. (2008)	37	W	M	6.85	8.50	1.65	8.05	8.05	0.00	9.06	10.53	1.47	7.65	8.88	1.24			
			SD	1.95	2.50	2.21	1.76	1.96	1.84	2.08	2.00	1.77	1.87	2.06	2.08			
2. Picture study, UM. Berman et al. (2008)	12	W	M	9.33	10.33	1.00	9.83	11.00	1.17	6.50	8.33	1.83	5.83	6.67	0.83			
			SD	3.78	2.66	1.79	3.54	2.00	1.83	2.26	3.14	1.60	2.48	2.50	3.06			
3. Walk, healthy sample, UM 2011.	21	W	M	7.16	8.89	1.73	9.27	10.27	1.00	9.50	10.25	0.75	7.58	9.33	1.75			
			SD	3.08	3.28	1.56	2.57	2.33	1.95	2.72	1.65	2.18	2.52	2.47	2.97			
4. Walk, MDD sample, UM. Berman et al. (2012)	19	W	M	7.11	8.00	0.89	9.00	8.00	−1.00	7.70	9.20	1.50	7.48	7.70	0.23			
			SD	3.02	2.55	1.45	1.94	1.41	1.00	3.13	3.16	1.43	2.79	2.87	0.67			
5. Picture study, UC, 2015.	45	W	M	8.57	9.29	0.71	9.43	8.86	−0.57	9.83	9.63	−0.21	8.67	9.17	0.50			
			SD	1.86	1.35	2.08	1.83	1.90	1.66	2.32	2.67	2.34	2.78	2.62	2.15			
6. Picture study, UM, 2015.	37	W	M	8.31	9.44	1.13	9.94	9.19	−0.75	8.86	9.19	0.33	8.29	9.19	0.90			
			SD	2.33	1.93	1.89	2.32	2.54	1.98	2.74	2.66	1.68	2.17	2.27	1.92			
7. Walk, UC, 2016.	49	W	M	8.96	9.50	0.54	9.58	9.12	−0.46	10.43	10.96	0.52	9.17	9.91	0.74			
			SD	2.52	2.90	2.14	2.61	2.45	1.58	3.63	3.20	2.87	2.64	3.36	2.32			
8. Virtual Reality study, UC, 2016.	82	W	M	8.65	9.45	0.80	9.85	10.00	0.15	10.71	11.19	0.48	9.50	10.55	1.05			
			SD	2.59	2.45	2.19	3.13	2.72	1.82	2.59	2.92	2.04	2.03	2.61	2.38			
9. Virtual Reality study- with habituation, UC, 2016.	82	B	M				10.40	9.85	−0.55	9.95	10.12	0.17				8.87	9.66	0.79
			SD				2.85	2.76	2.10	2.41	2.84	2.09				2.44	2.37	2.26
10. Composite study- Sounds, UC. Van Hedger et al. (2018)	44	B	M	9.77	10.64	0.86							8.50	8.77	0.27			
			SD	2.56	2.52	2.14							3.19	2.88	2.55			
11. Composite study- Pictures. UC, 2016	40	B	M	9.42	9.95	0.53							8.33	9.19	0.86			
			SD	2.67	2.20	2.25							2.58	2.64	2.20			
12. Video study, UBC. Bourrier et al. (2018)	60 (excl. 30 “other” controls)	B	M	7.53	8.83	1.30							7.73	7.80	0.07	7.17	7.70	0.53
			SD	2.78	2.52	2.05							3.17	3.31	2.85	3.17	2.79	2.39
13. Picture dose study- Session 1 & 2. UM, 2009.	39 (37 in 2nd session)	Only nature	M	6.15	7.18	1.03				7.68	8.14	0.46						
			SD	2.12	2.47	1.77				2.19	2.55	1.69						
**Total, W samples (study 1-8)**	302	W	M	8.24	9.21	0.97	9.42	9.28	−0.14	9.65	10.26	0.60	8.55	9.45	0.90			
			SD	2.57	2.45	2.04	2.56	2.42	1.80	2.89	2.84	2.16	2.48	2.76	2.22			
			N	149			149			153			153					
**Total, B samples (study 9-12)**	226	B	M	8.73	9.69	0.96	10.40	9.85	−0.55	9.95	10.12	0.17	8.14	8.49	0.36	8.41	9.13	0.72
			SD	2.84	2.53	2.13	2.85	2.76	2.10	2.41	2.84	2.09	3.00	3.02	2.57	2.75	2.63	2.29
			N	71			40			42			73			112		
**Grand Total**	**567**		M	8.06	9.04	0.98	9.62	9.40	−0.23	9.39	9.89	0.50	8.41	9.14	0.72	8.41	9.13	0.72
			SD	2.71	2.59	2.02	2.65	2.50	1.87	2.80	2.89	2.08	2.66	2.87	2.35	2.75	2.63	2.29
			N	259			189			232			226			112		

*M, mean; SD, standard deviation. UM, Univ. of Michigan; UC, Univ. of Chicago; UBC, Univ. Of British Columbia; W, environment is a within-subjects factor; B, environment is a between-subjects factor*.

### Review of Other Studies in the Literature

To compare the findings from our own studies with those of others, a review of the literature and summary of findings was performed for existing randomized controlled or randomized crossover studies that tested the effects of nature vs. urban or control exposures on BDS performance, as well as on other executive cognitive performance tests. The same inclusion/exclusion criteria were employed when reviewing the literature as that for the studies from our own lab's pooled data analyses. As such, only randomized controlled studies or randomized controlled crossover studies measuring cognitive performance both pre- and post-environment exposure were included. Study populations included students (undergraduate and graduate students) and non-student adults. Since the data-analyses and results presented in this paper concern effects on BDS, the focus of the literature review was also primarily on findings for BDS. These findings were documented in a table including descriptive statistics for BDS performance for each environment × time cell, and/or pre-post change scores (depending on the reported information). In addition, study findings utilizing other cognitive tests were also reviewed and were summarized in terms of the test statistics reported in those papers to provide an overview for comparison to the BDS effects.

The literature search was performed using multiple search engines for academic journal articles and reports, including Google Scholar, PubMed, Scopus, and PsychInfo. Searches were concluded by the end of March 2017. Combinations of the following keywords were used in the literature searches: *nature, natural, natural environments, biophilia, attention, cogni*t^*^, restoration. Reference sections of the obtained papers were examined for additional studies, and a descendancy search was also conducted for studies that cited the obtained papers. The review of the literature may however not be exhaustive.

## Results From the Studies on BDS and Pooled Data-Analyses

**Figure 1:**
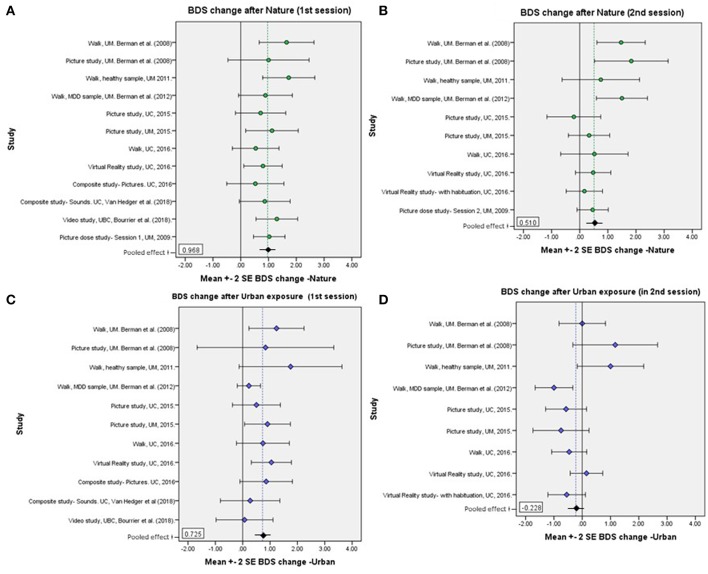
**(A–D)** Mean BDS score changes pre- to post- nature vs. urban environment interactions, by order of environment conditions, for each study sample. **(A)** BDS change after nature, 1st sessions; **(B)** BDS change after nature, 2nd sessions; **(C)** BDS change after urban exposure, 1st sessions; **(D)** BDS change after urban exposures, 2nd sessions. The dotted reference lines represent the grand mean of all participants included in the respective plots/conditions. See results in Table 3.

**Figure 2:**
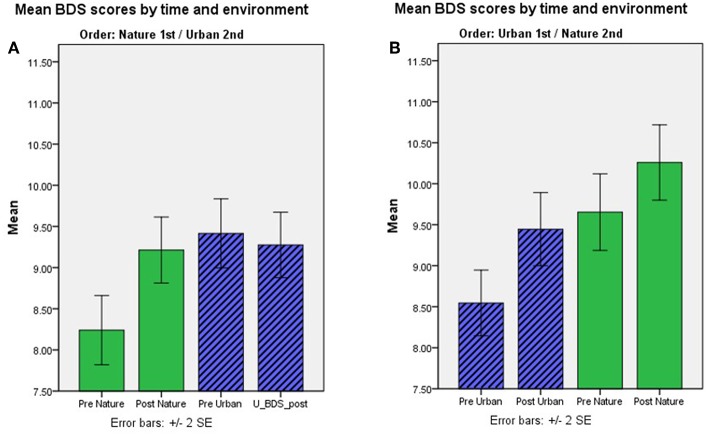
**(A,B)** BDS performance scores pre- and post-nature vs. urban environment interactions, by order of environment conditions. Aggregated results for study samples with environment as a within-subjects factor. Mean BDS scores by time and environment condition, for the order of conditions nature 1st and urban 2nd; **(A)** and urban 1st and nature 2nd **(B)**.

Mean BDS scores by time, environment condition, and order, for each study sample, are shown in Table 2, and changes in BDS performance scores after nature vs. urban/non-nature interactions, by session order, for each study, are illustrated in Figures 1A–D. Furthermore, aggregated BDS scores for the studies with environment as a within-subjects factor are shown by time, environment, and order in Figure 2. Paired samples t-statistics for BDS change from pre- to post nature vs. urban environment interactions in the 1st vs. the 2nd test sessions, *F*-statistics for time (pre, post) by environment (nature, urban) interactions, and time by environment by order interactions, are shown in Table 3. BDS change scores, *t*-statistics, and 95% confidence intervals across the studies, from the nature vs. urban environment conditions, separated by whether the environment condition occurred in the 1st vs. the 2nd test sessions, are also shown in Table 3.

**Table 3:** Effects on BDS performance from pre- to post environment exposures by study sample, and aggregated time (pre- vs. post environment) by environment conditions (nature vs. urban) by order effects on BDS across study samples.

**Study sample**			**Nature 1st/Urban 2nd**		**Urban 1st/Nature 2nd**		**Other**		Time^*^ Env		Time ^*^ Env ^*^ Order	
	**N**	***t-test***	**Pre-post Nature (S1)**	**Pre-post Urban (S2)**	**Pre-post Nature (S2)**	**Pre-post Urban (S1)**	**Pre-post (S1)**	***F*-test**	***F***	***$Eta_{p}^{2}$***	***F***	***$Eta_{p}^{2}$***
1. Walk, UM, Berman et al. (2008)	37	t	**3.343**	0.000	**3.422**	**2.452**		F	**5.670**	0.139	3.193	0.084
		df	19	19	16	16		df	1, 35		1, 35	
		p	0.003	1.000	0.003	0.026		p	0.023		0.083	
2. Picture study, UM, Berman et al. (2008)	12	t	1.369	1.557	**2.803**	0.667		F	0.486	0.046	0.953	0.087
		df	5	5	5	5		df	1, 10		1, 10	
		p	0.229	0.180	0.038	0.534		p	0.501		0.352	
3. Walk, healthy sample, UM, 2011.	21	t	**3.684**	1.701	1.090	1.861		F	0.033	0.002	1.313	0.065
		df	10	10	9	9		df	1, 19		1, 19	
		p	0.004	0.120	0.304	0.096		p	0.858		0.266	
4. Walk, MDD sample, UM, Berman et al. (2012)	19	t	1.835	**−3.000**	**3.308**	1.060		F	**18.661**	0.523	0.703	0.040
		df	8	8	9	9		df	1, 17		1, 17	
		p	0.104	0.017	0.009	0.317		p	0.000		0.414	
5. Picture study, UC, 2015.	45	t	1.576	−1.577	−0.436	1.141		F	0.423	0.010	**5.049**	0.105
		df	20	20	23	23		df	1, 43		1, 43	
		p	0.131	0.130	0.667	0.266		p	0.519		0.030	
6. Picture study, UM, 2015.	37	t	**2.377**	−1.513	0.907	**2.158**		F	1.844	0.050	**6.495**	0.157
		df	15	15	20	20		df	1, 35		1, 35	
		p	0.031	0.151	0.375	0.043		p	0.183		0.015	
7. Walk, UC, 2016.	49	t	1.283	−1.489	0.871	1.528		F	0.610	0.013	1.476	0.030
		df	25	25	22	22		df	1, 47		1, 47	
		p	0.211	0.149	0.393	0.141		p	0.439		0.230	
8. Virtual Reality 1, UC, 2016.	82	t	**2.314**	0.521	1.513	**2.855**		F	0.015	0.000	3.621	0.043
		df	39	39	41	41		df	1, 80		1, 80	
		p	0.026	0.605	0.138	0.007		p	0.903		0.061	
9. Virtual Reality 2- with habituation, UC, 2016.	82	t		−1.657	0.516		**3.169**	F	2.393	0.029		
		df		39	41		81	df	1, 80			
		p		0.106	0.609		0.002	p	0.126			
10. Composite study- Sounds, UC, Van Hedger et al. (2018)	44	t	1.889			0.502		F	0.692	0.016		
		df	21			21		df	1, 42			
		p	0.073			0.621		p	0.410			
11. Composite study- Pictures, UC, 2016.	40	t	1.022			1.788		F	0.221	0.006		
		df	18			20		df	1, 38			
		p	0.320			0.089		p	0.641			
12. Video study, UBC, Bourrier et al. (2018)	60 (+30)	t	**3.467**			0.128	1.223	F	3.695	0.060		
		df	29			29	29	df	1, 58			
		p	0.002			0.899	0.231	p	0.059			
13. Picture dose study- Session 1 and 2, UM, 2009.	39	t	**3.620**		1.651			F				
		df	38		36			df				
		p	0.001		0.107			p				
**Total, studies with env as within-subj factor** (study 1–8)	302	t	**5.833**	−0.955	**3.467**	**5.017**		F	**5.989**	0.020	**17.78**	0.056
		df	148	148	152	152		df	1, 300		1, 300	
		p	0.000	0.341	0.001	0.000		p	0.015		0.000	
**Total, studies with env as betw-subj factor** (study 9–12)	226	t	**3.793**	−1.657	0.516	1.183	**3.341**	F	**4.223**	0.019		
		df	70	39	41	72	111	df	1, 224			
		p	0.000	0.106	0.609	0.241	0.001	p	0.041			
**Grand Total**	**528^‡^**	t	**6.966**	−1.673	**3.320**	**4.64**						
		df	219	188	194	225						
		p	0.000	0.096	0.001	0.000						
		M diff	0.968	−0.228	0.510	0.725						
		SE	0.139	0.136	0.154	0.156						
	95% CI	Lower	0.694	−0.496	0.207	0.417					
		Upper	1.242	0.041	0.813	1.032						

F-statistics are from factorial general linear models with BDS as the dependent variable and the factors time

* environment, and additionally time^*^environment^*^order for within-subjects study designs. Time is always a within-subjects factor. Environment is a within-subjects factor in the randomized crossover studies, and a between-subjects factor in the randomized controlled studies. Order of environment conditions is a between-subjects factor in the randomized crossover studies. M diff = mean difference between BDS performance scores pre- vs. post the environment interaction. Statistically significant (p < 0.05) t-values and F-values are marked in bold. UM, Univ. of Michigan; UC, Univ. of Chicago; UBC, Univ. of British Columbia; S1, test session 1; S2, test session 2.

‡ *Excludes the Picture dose study sample*.

### Results for Studies With Environment as a Within-Subjects Factor—Time × Environment and Time × Environment × Order Effects on BDS Performance and Affect

#### Time × Environment Effects on BDS

The results from the ANOVA (Table 4, section A) showed that there was a significant time × environment interaction effect on BDS (*p* = 0.021), whereby BDS performance improved more after interacting with nature compared to urban environments. There was also a large main effect of time on BDS (*p* < 0.001), whereby performance markedly improved from pre- to post-environment interactions.

**Table 4:** Results from ANOVAs on time (pre, post) × environment (nature, urban) effects (A), and time × environment × order (nature 1st, urban 1st) effects (B), on BDS, for studies with environment as a within-subjects factor.

**A. TIME (PRE, POST)** * **ENVIRONMENT (NATURE, URBAN) EFFECTS ON BDS**						
**Source**	**Type III sum of squares**	**df**	**Mean square**	**F**	***p***	**${\text{η}}_{\text{p}}^{2}$**
*Within-subjects contrasts:*						
Environment	9.880	1	9.880	2.691	0.102	0.009
Error(Environment)	1105.135	301	3.672			
Time	103.885	1	103.885	48.069	0.000	0.138
Error(Time)	650.506	301	2.161			
Environment ^*^ Time	12.070	1	12.070	**5.418**	**0.021**	0.018
Error(Environment ^*^Time)	670.571	301	2.228			
**B. TIME (PRE, POST)** ^*^ **ENVIRONMENT (NATURE, URBAN)** ^*^ **ORDER (NATURE 1ST, URBAN 1ST**) **EFFECTS ON BDS**						
*Within-subjects contrasts:*						
Environment	8.769	1	8.769	2.869	0.091	0.009
Environment ^*^ Order	188.229	1	188.229	61.586	0.000	0.170
Error(Environment)	916.906	300	3.056			
Time	103.079	1	103.079	48.170	0.000	0.138
Time ^*^ Order	8.540	1	8.540	3.991	0.047	0.013
Error(Time)	641.966	300	2.140			
Environment ^*^ Time	12.638	1	12.638	**5.989**	**0.015**	0.020
Environment ^*^ Time ^*^ Order	37.511	1	37.511	**17.776**	**0.000**	0.056
Error(Environment ^*^Time)	633.060	300	2.110			
*Between-subjects contrasts:*						
Intercept	103475.168	1	103475.168	5094.677	0.000	0.944
Order	58.410	1	58.410	2.876	0.091	0.009
Error	6093.135	300	20.310			

*The results for the time × environment interaction and the time × environment × order interaction are marked in bold*.

#### Time × Environment × Order (Nature or Urban Condition 1st) Effects on BDS

BDS performance scores pre- and post-nature vs. urban environment interactions, by order of environment conditions, are shown in Figure 2. The results from the ANOVA testing the effects of time (pre, post) × environment (nature, urban) × order of conditions (nature 1st, urban 1st) on BDS showed a significant time × environment interaction effect (*p* = 0.015), and a strong time × environment × order (of environment conditions) interaction effect (*p* < 0.001), on BDS performance (Table 4, section B). Specifically, BDS performance improved after both nature (*p* < 0.001) and urban (*p* < 0.001) environment conditions *when tested in the first session* (Table 3). When *tested in the second session*, significant improvements in BDS performance occurred only after interacting with nature (*p* < 0.001), and not after interacting with urban (*p* > 0.05) environments (Table 3). See Tables 2, 3, and Figures 1A–D, for a summary of these effects.

In sum, in the studies with a RCrT design (i.e., with environment as a within-subjects factor), there was a significant time × environment interaction, whereby BDS performance improved more after nature environmental interactions than after urban environmental interactions. There was also a strong time × environment × order interaction, whereby the pattern of performance changes after nature vs. urban interactions differed depending on the order in which participants experienced the environmental conditions. Specifically, BDS performance improved only after nature but not urban interactions if tested in the second session (after initial practice effects occurred in the 1st test sessions).

#### Time × Environment × Order Effects on Positive Affect and Negative Affect

Descriptive statistics for positive affect (PA) and negative affect (NA) are shown in Table 5. Results from the ANOVA on PA (Table 6, section A) showed that there was no significant main effect of time, but a clear time × environment effect (*p* < 0.001), and a time × environment × order effect (*p* < 0.001), whereby PA increased after nature but not after urban interactions and was only present when the nature condition was administered in the second session. For NA (Table 6, section B), there was a main effect of time (*p* < 0.001), whereby NA decreased across both environment conditions, but no significant time × environment interaction effect (*p* = 0.211), nor time × environment × order interaction effect (*p* = 0.552).

**Table 5:** Descriptive statistics for Positive and Negative Affect, for study samples with environment as a Within-subjects factor, by the order in which the environment conditions were administered.

			**Positive affect**			**Negative affect**		
**Order**	**Environment**	**Time**	***N***	**Mean**	**SD**	***N***	**Mean**	**SD**
Nature 1st /Urban 2nd	Nature	Pre	145	2.610	0.760	139	1.458	0.536
		Post	145	2.563	0.846	139	1.306	0.428
	Urban	Pre	145	2.347	0.773	139	1.463	0.611
		Post	145	2.296	0.764	139	1.375	0.504
Urban 1st /Nature 2nd	Nature	Pre	152	2.357	0.849	148	1.516	0.642
		Post	152	2.534	0.869	148	1.362	0.555
	Urban	Pre	152	2.663	0.783	148	1.576	0.653
		Post	152	2.547	0.867	148	1.445	0.586

**Table 6:** Results from ANOVAs on time (pre, post) × environment (nature, urban) × order (nature 1st, urban 1st) effects on positive affect and negative affect, for study samples with environment as a Within-subjects factor.

**A. RESULTS FOR POSITIVE AFFECT**						
**Dependent measure: Positive affect**						
**Source**	**Type III sum of squares**	**df**	**Mean square**	***F***	***p***	**ηp2**
*Within-subjects contrasts:*						
Time	0.026	1	0.026	0.061	0.805	0.000
Time ^*^ Order	0.466	1	0.466	1.093	0.297	0.004
Error(time)	125.668	295	0.426			
Environment	0.818	1	0.818	2.605	0.108	0.009
Environment ^*^ Order	13.385	1	13.385	42.649	0.000	0.126
Error(Environment)	92.581	295	0.314			
Time ^*^ Environment	1.643	1	1.643	**13.684**	**0.000**	0.044
Time ^*^ Environment ^*^ Order	1.553	1	1.553	**12.933**	**0.000**	0.042
Error(time^*^Environment)	35.413	295	0.120			
*Between-subjects effects:*						
Intercept	7358.059	1	7358.059	4083.300	0.000	0.933
Order	1.505	1	1.505	0.835	0.362	0.003
Error	531.587	295	1.802			
**B. RESULTS FOR NEGATIVE AFFECT**						
**Dependent measure: Negative affect**						
*Within-subjects contrasts:*						
Time	4.921	1	4.921	28.215	0.000	0.090
Time ^*^ Order	0.036	1	0.036	0.207	0.649	0.001
Error(time)	49.707	285	0.174			
Environment	0.852	1	0.852	5.274	0.022	0.018
Environment ^*^ Order	0.087	1	0.087	0.541	0.463	0.002
Error(Environment)	46.028	285	0.162			
Time ^*^ Environment	0.129	1	0.129	**1.574**	**0.211**	0.005
Time ^*^ Environment ^*^ Order	0.029	1	0.029	**0.355**	**0.552**	0.001
Error(time^*^Environment)	23.380	285	0.082			
*Between-subjects effects:*						
Intercept	2370.513	1	2370.513	2687.872	0.000	0.904
Order	1.582	1	1.582	1.794	0.181	0.006
Error	251.350	285	0.882			

*The results for the time × environment interaction and the time × environment × order interaction are marked in bold*.

#### Interaction Effects on BDS, Adjusting for Positive Affect, and Negative Affect

Since there was a clear time × environment effect, and a clear time × environment × order effect on both BDS and PA, we wanted to examine if these interaction effects on BDS performance were preserved after adjusting for PA by including pre- and post-environment affect as a time-varying covariate in the factorial model, computed using the Linear Mixed Models procedure in SPSS. Although effects on NA were not significant, we used the recommended dropping rule of *p* < 0.250, and thus also performed the analysis adjusting for NA as a time-varying covariate. The results showed that adjusting for PA did not cause much change in the time × environment effect (*p* = 0.017), nor the time × environment × order effect (*p* < 0.001), on BDS performance (Table 7, section A), compared to the results without adjustment for PA (Table 4). Similarly, adjusting for NA did not cause much change in the time × environment effect (*p* = 0.016), nor the time × environment × order effect (*p* < 0.001), on BDS performance (Table 7, section B), compared to the results without adjustment for NA. Supplementary Material regarding these analyses and results are given in Appendix A in Supplementary Material, section 2.

**Table 7:** Effects^†^ of time (pre, post) × environment (nature, urban) × order (nature 1st, urban 1st) effects on BDS, adjusting for Positive Affect vs. Negative Affect, for study samples with environment as a within-subjects factor.

**A. MODEL RESULTS ADJUSTING FOR POSITIVE AFFECT**				
**Source**	**Numerator df**	**Denominator df**	***F***	***p***
Intercept	1	957.885	1271.581	0.000
Time	1	299.778	47.996	0.000
Environment	1	301.913	2.635	0.106
Order	1	302.157	2.705	0.101
Time ^*^ Environment	1	304.091	**5.731**	**0.017**
Time ^*^ Order	1	300.274	3.668	0.056
Environment ^*^ Order	1	316.371	64.152	0.000
Time ^*^ Environment ^*^ Order	1	304.323	**18.843**	**0.000**
PA	1	975.600	2.726	0.099
**B. MODEL RESULTS ADJUSTING FOR NEGATIVE AFFECT**				
Intercept	1	863.728	1826.778	0.000
Time	1	302.952	39.289	0.000
Environment	1	292.778	2.402	0.122
Order	1	291.737	1.842	0.176
Time ^*^ Environment	1	291.335	**5.822**	**0.016**
Time ^*^ Order	1	289.657	3.605	0.059
Environment ^*^ Order	1	291.268	56.301	0.000
Time ^*^ Environment ^*^ Order	1	291.029	**17.468**	**0.000**
NA	1	974.437	1.477	0.225

† *Type III Tests of Fixed Effects. Dependent measure: BDS score*.

*The results for the time × environment interaction and the time × environment × order interaction are marked in bold*.

In sum, for these studies with environment as a within-subjects factor, there were interaction effects of time × environment and time × environment × order on PA but not on NA. PA increased after nature interactions but not after urban interactions, but this effect was restricted to the second sessions. Importantly, the time × environment and time × environment × order effects on BDS were hardly changed after adjusting for either PA or NA.

### Results for Study Samples With Environment as a Between-Subjects Factor—Time × Environment Effects on BDS Performance and Affect

An ANOVA was used to test the effects of nature vs. urban environment interactions in the studies implementing a RCT design (i.e., environment was a between-subjects factor).

#### Time × Environment Effects on BDS

The results showed that there was a significant time × environment interaction effect on BDS performance (*p* < 0.05), whereby BDS performance improved more after nature compared with urban environments, and a main effect of time (*p* < 0.05), whereby BDS performance improved from pre- to post environment exposures (Table 8).

**Table 8:** Results from ANOVAs on time (pre, post) × environment (nature, urban) effects on BDS, for studies with environment as a between-subjects factor.

**Source**	**Type III sum of squares**	**df**	**Mean square**	***F***	***p***	**${\text{η}}_{\text{p}}^{2}$**
*Within-subjects effects:*						
Time	13.808	1	13.808	5.229	0.023	0.023
Time ^*^ Environment	11.153	1	11.153	**4.223**	**0.041**	0.019
Error(pre_post)	591.540	224	2.641			
*Between-subjects effects:*						
Intercept	38563.330	1	38563.330	2758.494	0.000	0.925
Environment	35.684	1	35.684	2.553	0.112	0.011
Error	3131.487	224	13.980			

*The results for the time × environment interaction and the time × environment × order interaction are marked in bold*.

#### Time × Environment Effects on Positive Affect and Negative Affect

Descriptive statistics for PA and NA are shown in Table 9. The analyses of PA and NA exclude the Video study from UBC (Bourrier et al., 2018) because affect was not measured in this study.

**Table 9:** Descriptive statistics for Positive and Negative Affect, for study samples with environment as a between-subjects factor.

		***N***	**Positive affect**		**Negative affect**	
**Environment**	**Time**		**Mean**	**SD**	**Mean**	**SD**
Nature	Pre	83	2.788	0.700	1.678	0.643
	Post	83	2.625	0.857	1.502	0.586
Urban	Pre	83	2.911	0.863	1.604	0.681
	Post	83	2.622	0.918	1.547	0.707

The results from the ANOVA on PA showed that there was a main effect of time (*p* < 0.001), whereby PA decreased in both environment conditions, but no significant time × environment interaction effect (*p* = 0.185) on PA (Table 10, section A). For NA, there was a main effect of time (*p* < 0.001), whereby NA decreased in both environment conditions, and a trending time × environment interaction on NA (*p* = 0.076), whereby NA tended to decrease more after nature than after urban exposures (Table 10, section B).

**Table 10:** Results from ANOVAs on time (pre, post) × environment (nature, urban) effects on Positive vs. Negative Affect, for study samples with environment as a between-subjects factor.

**A. RESULTS FOR POSITIVE AFFECT**						
**Dependent Measure: Positive affect**						
**Source**	**Type III sum of squares**	**df**	**Mean square**	***F***	***p***	${\text{η}}_{\text{p}}^{2}$
*Within-subjects contrasts:*						
Time	4.236	1	4.236	22.644	0.000	0.121
Time ^*^ Environment	0.332	1	0.332	1.775	0.185	0.011
Error(Time)	30.677	164	0.187			
*Between-subjects effects:*						
Intercept	2486.061	1	2486.061	2040.112	0.000	0.926
Environment	0.295	1	0.295	0.242	0.623	0.001
Error	199.849	164	1.219			
**B. RESULTS FOR NEGATIVE AFFECT**						
**Dependent Measure: Negative affect**						
*Within-subjects contrasts:*						
Time	1.122	1	1.122	12.115	0.001	0.069
Time ^*^ Environment	0.295	1	0.295	3.188	0.076	0.019
Error(Time)	15.188	164	0.093			
*Between-subjects effects:*						
Intercept	831.778	1	831.778	1083.592	0.000	0.869
Environment	0.019	1	0.019	0.025	0.876	0.000
Error	125.888	164	0.768			

*These analyses exclude the Video study from UBC (Bourrier et al., 2018), since affect was not measured in this study*.

Since the analyses on affect excluded the video study from UBC (as this study did not measure affect), the time × environment effect on BDS was also computed after excluding this study, to compare these results with those adjusted for affect. The results showed that there was no longer a significant time × environment interaction effect on BDS, *F*_(1, 164)_ = 1.413, *p* = 0.236, after excluding the video study from UBC. Therefore, additional models testing time × environment effects on BDS after adjustment for PA and NA were not performed separately for this subset of studies with an RCT design. In sum, in the studies with an RCT design (i.e., with environment as a between-subjects factor), there was a significant time × environment effect on BDS performance, whereby performance improved more after the nature exposures compared with the urban exposures. Unlike in the within-subject designs, there was no significant time × environment effect on PA, nor on NA.

### Time × Environment Effects on BDS, Stratified by 1st vs. 2nd Test Sessions, Including All Study Samples

Since the effects of the order in which environment conditions were administered were significant in predicting BDS performance changes in the studies implementing a RCrT design, we also analyzed the environment × time effects on BDS separately for all first and second sessions. That is, these analyses included data from the studies which tested environment as a within- or between-subjects factor, comparing the effects of nature vs. urban conditions on BDS in all first sessions, vs. in all second sessions (see Table 11). In order to compare the effects on BDS with and without adjustment for affect, the linear mixed effects model procedure was used in SPSS to compute the factorial models (fixed effects models) with environment as a between-subjects factor and time (pre- vs. post exposure) as a within-subjects factor. Maximum likelihood estimation was used and unstructured covariance type for the repeated factor. For data from the first test sessions (*N* = 446), the time by environment interaction was not significant (*p* = 0.244), while the main effect of time (pre, post) was large (*p* < 0.001), where performance improved from pre- to post-test across environment conditions (i.e., practice effects; see Table 11). For data from the second test sessions (*N* = 384), devoid of initial practice effects, the time by environment interaction was very robust (*p* < 0.001), whereby BDS performance improved more after nature than urban exposures (Table 11). The main effect of time (pre, post) in second sessions became non-significant (*p* = 0.70, Table 11). Again, these results illustrate the importance of delineating the effects of practice (primarily occurring in first sessions, as evidenced by the large main effect of time across the environment conditions in the first but not in the second sessions) in order to adequately evaluate the effects of the environment exposures (in the second sessions) on cognitive performance.

**Table 11:** Effects^†^ of time (pre, post) × environment (nature, urban) on BDS, in 1st vs. 2nd test sessions, including all study samples.

**Session**	**Source**	**Numerator df**	**Denominator df**	***F***	***p***
1	Intercept	1	446	5894.507	0.000
	Time	1	446	65.624	0.000
	Environment	1	446	0.216	0.642
	Time ^*^ Environment	1	446	1.359	0.244
2	Intercept	1	384	5850.396	0.000
	Time	1	384	1.900	0.169
	Environment	1	384	3.297	0.070
	Time ^*^ Environment	1	384	12.936	0.000

† *Type III Tests of Fixed Effects. Dependent measure: BDS score*.

**Diagram 1:**
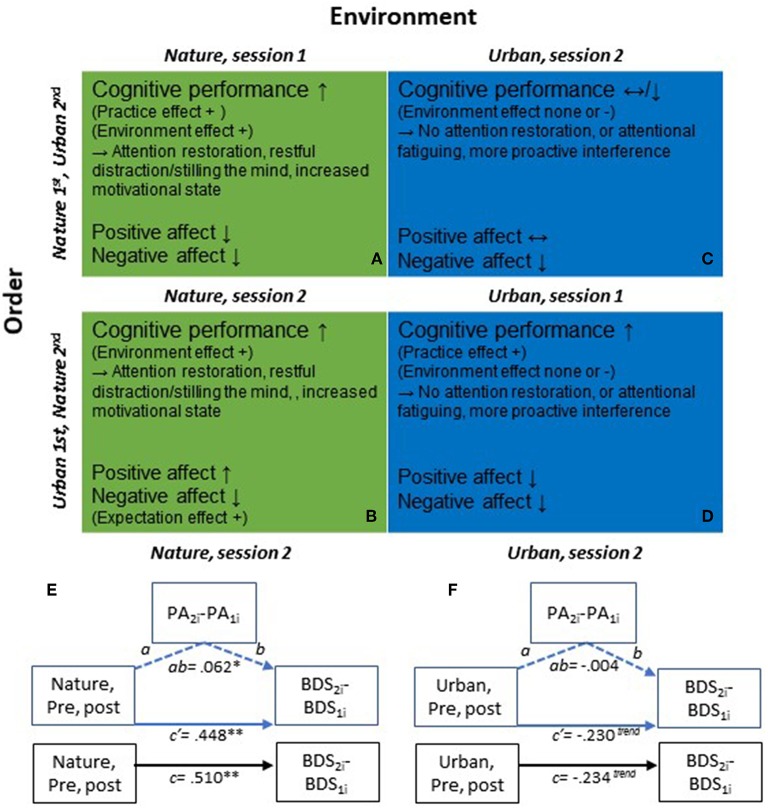
**(A–D)** Illustration of general effects and potential mechanisms of action on cognitive performance and affect from pre- to post nature vs. urban environment interactions, and the order of participation in the environment conditions. **(A)** Nature environment interaction, Nature condition is tested in session 1; **(B)** Nature environment interaction, Nature condition is tested in session 2; **(C)** Urban environment interaction, Urban condition is tested in session 2; **(D)** Urban environment interaction, Urban condition is tested in session 1. Depending on the order in which individuals participate in the nature and urban conditions, the changes observed on cognitive performance and affect also differ. In first test sessions **(A,D)**, there are clear practice effects on cognitive performance in addition to the effects that are caused by the environment condition *per se*. Second test sessions **(B,C)** are instead devoid of the initial performance improvement due to practice, and the cognitive performance improvements observed constitute more clean effects of the environment conditions. Thus, the second sessions provide a better evaluation of the effects of the environment conditions *per se* on cognitive performance. Changes in affect also differ depending on the order of environment conditions. That is, positive affect increased after nature interactions performed in the second but not first sessions, which could be due to different expectations on the second session, depending on the experience (environment condition) in the prior, first session. That is, if the urban condition was done first, the expectations on the second condition may be low and the actual experience after the environment interaction in session 2 may have exceeded expectations. **(E,F)** Mediational path analyses of total, direct & indirect effects on BDS performance, via positive affect (PA), following nature vs. urban environment interactions, in 2nd test sessions. **(E)** Nature condition, 2nd test sessions: Total effect, c: 0.510 (*SE: 0.154, t* = *3.320, df* = *194, 95% CI: 0.207, 0.813*); Direct effect, c′ : 0.448 (*SE: 0.155, t* = *2.885, df* = *192, 95% CI: 0.142, 0.755*); Indirect effect, ab: 0.062 (*SE: 0.038, 95% CI: 0.001, 0.145*). **(F)** Urban condition, 2nd test sessions: Total effect, *c*: −0.234 (*SE: 0.137, t* = −*1.714, df* = *187, 95% CI:* −*0.504, 0.035*); Direct effect, c′ : −0.230 (*SE: 0.138, t* = −*1.670, df* = *185, 95% CI:* −*0.503, 0.042*); Indirect effect, *ab*: −0.004 (*SE: 0.019, 95% CI:* −*0.050, 0.032*). ^**^p < *0.01*, ^*^p < *0.05*, ^*trend*^*p* < *0.10*.

The significant time by environment interaction effect in the second sessions did not change much after adjusting for PA, *F*_(1, 387.8)_ = 11.375, *p* < 0.001, or adjusting for NA, *F*_(1, 373.9)_ = 12.099, *p* < 0.001 (Appendix A, Supplementary Tables 11.2 and 11.3). Based on the marked practice effects observed on the BDS across environment conditions tested in the 1st sessions, a potential relationship between changes in BDS and changes in affect due to the environment interaction should be best evaluated when tested in the 2nd session and in conditions where there is a significant change in BDS and/or affect (i.e., primarily in the nature conditions). In line with this reasoning, BDS changes and affect changes were not correlated in any other environment × session order cell, except in the nature condition tested in the 2nd session. However, only a weak correlation was observed between pre- to post-nature BDS change and PA change (*r* = 0.148*, p* = 0.038, Supplementary Table 11.4), and thus changes in PA could only explain 2.2 % of the changes in BDS (r^2^ = 0.0219). There was no observed correlation between BDS change and NA change. Supplementary Material regarding these analyses and results are given in Appendix A in Supplementary Material, section 2.2.4. To further evaluate any potential mediation of BDS changes through PA changes, after nature interactions in the second test sessions, supplementary mediational path analyses were also performed (see Appendix C in Supplementary Material). The results of the path analyses are presented in Diagram 1. E-F and in Appendix C in Supplementary Material, and showed that out of the total improvement of 0.510 BDS points after the nature interaction, only 0.062 points of this improvement could be mediated by positive affect. These results further indicate that the positive effect of nature interactions (i.e., in 2nd sessions, devoid of initial practice effects), observed on BDS, could not be explained by changes in PA or NA.

## Results From Reviewing the Literature

Studies from outside of our laboratories and our collaborators laboratories were reviewed that employed a RCT or RCrT design to test the effects of nature exposures, compared to urban or other control conditions, on BDS performance or other executive cognitive performance tasks evaluated pre- and post the environment exposure conditions.

Twenty-seven studies were identified in the literature which met the inclusion criteria and the characteristics and results of these are summarized in Supplementary Table 12 in Appendix B.

Out of these 27 studies, 12 studies included BDS measurement pre- and post the environmental exposure conditions. Mean BDS scores pre- and post each environmental exposure (for different subgroups, where applicable) are reported in Supplementary Table 13 in Appendix B (if reported in the original reports). Five of these 12 studies reported a statistically significant effect of environment condition on BDS performance in favor of the nature condition (Cimprich and Ronis, 2003; Lin et al., 2014; Rogerson and Barton, 2015; Gidlow et al., 2016; Li and Sullivan, 2016), one of which may not have found a significant time by environment interaction, since only post-intervention scores were reported (Cimprich and Ronis, 2003). The other 7 studies reported no favorable effects of the nature conditions on BDS performance (Bodin and Hartig, 2003; Stark, 2003; Perkins et al., 2011; Emfield and Neider, 2014; Gamble et al., 2014; Bratman et al., 2015a; Triguero-Mas et al., 2017).

The results for environment effects on BDS in the 12 different studies identified in the literature review were heterogeneous. Importantly, these studies did not parcel out practice effects from environment effects. The varying findings from these studies could thus stem from practice effects contaminating the effects of the environmental condition to varying degrees in the different studies. This is especially true for those studies employing an RCT design such that all test sessions are likely contaminated by practice effects. In addition, the heterogeneity of these results could also stem from other differences in the experimental designs used, sample sizes, experimental procedures and experimental interventions/stimuli.

For example, Bratman et al. (2015a) and Gamble et al. (2014) (who also used the same BDS measure as in our own studies) did not find any significant time by environment effects on BDS, but found improvements from pre- to post exposure in both environment conditions. However, these studies also both employed a RCT design, which means that all test sessions were first sessions and were thus very likely to be confounded by practice effects. Gable et al. reported practice effects with a mean BDS improvement of 0.87 BDS scores in both the nature and urban conditions.

To compare with the results across the studies from Berman and colleagues, the difference between the nature and urban conditions regarding changes in BDS performance were clearly smaller in the first sessions than in the second sessions. In the first sessions, mean changes in BDS performance were 0.97 (*SD* = 2.06) scores for the nature conditions and 0.72 (*SD* = 2.35) for the urban conditions. The mean changes in BDS performance in the second sessions, devoid of initial practice effects, were instead 0.51 (*SD* = 2.15) after nature exposures and −0.23 (*SD* = 1.87) after urban environment exposures (Table 3). Considering the clear order/practice effects found in our analyses, such effects were also likely to be present in these studies and potentially confounded these results from these other laboratories. As such, the effects of nature compared to urban interactions on BDS performance improvements could be underestimated, and the statistical power reduced, in those studies due to such methodological limitations.

Regarding the effects of nature exposures on other executive cognitive tests, 17 different cognitive tests of executive and attentional processes (some including multiple performance measures) were used in addition to the forward and backward digit span, across the 27 studies that were identified in the literature (28 studies when including Berman et al.'s (2008) results on the Attention Network Task, ANT) (see Supplementary Table 12). These studies included the following tests, *with the number of studies using the test indicated within brackets*: ANT (with different test components assessing executive (4), orienting (3) or alerting attention (2)), Necker cube pattern control (NCPC (6)), Sustained attention to response task (SART (3)), Trail making test A (TMTA (2)) and B (TMTB (3)), Operation span (Ospan (1)), Symbol digit modalities test (1), Change detection (1), Logical memory (1), Error scale (1), Category matching (1), Colored number pictures (1), Reading span task (RST (2)), Stroop (1), Search and Memory test (1), Proofreading task (1), and Symbol substitution test (1).

While some reported significant performance improvements or superior performance post exposure in the nature conditions compared to the control conditions, only a few studies reported statistically significant *interactions* between time (pre-post environment exposure) and environment conditions (nature, urban, and/or other) in favor of the nature condition (see Supplementary Table 13). These include the single study testing Ospan (Bratman et al., 2015a), 2 out of 4 studies testing ANT-E (Berman et al., 2008; Gamble et al., 2014), 2 out of 4 studies testing NCPC (Hartig et al., 2003; Greenwood and Gatersleben, 2016), and the single study testing Colored number pictures (Chen et al., 2011). The latter found greater performance improvements after viewing nature pictures compared to day-time city pictures but not compared to viewing nightscape city pictures. The diverse findings on NCPC and ANT-E may also suggest that these tests do not optimally capture the effects that nature exposures might have on directed attention processes (possibly due to not being demanding enough on directed-attention processes, or having weaker reliability). However, null findings may also be related to study designs rather than the cognitive tasks employed, such as limited effectiveness and contrast between the environment conditions tested, limited sample sizes and power, varied testing procedures, and confounding effects of test practice and order effects in studies employing a crossover design with the environment conditions being a within-subjects factor. For example, Bratman et al. (2015a) used an extensive cognitive test battery and administered the cognitive tests in a randomized order for each participant but kept a similar order pre- and post-environment exposure. This design choice could abolish time by environment effects on some tests (like ANT-E and BDS) due to fatigue affecting performance on different tests for different participants both pre- and post the environment exposures and thereby confounding the assessment of cognitive performance changes invoked by the environment exposures. The significant effect on Ospan despite this test randomization procedure and long testing session, observed in the same study (Bratman et al., 2015a), may indicate that this type of measure is particularly sensitive to the effects of nature interactions. In fact, Ospan is also the task that is most similar to the BDS tasks in that both are demanding WM tasks that heavily tap several cognitive control processes dependent on executive attention. Both tasks require the maintenance of multiple items in short-term memory, updating, and also have a simultaneous processing component (reversing digit order in BDS and evaluating logical/mathematical statements in Ospan). Based on the findings of both the studies reported in this paper and other studies identified in the literature, such WM tasks seem to capture at least some of the effects that different environment exposures have on cognition, which was also observed by Stevenson et al. (2018). The evidence base is weaker for the other cognitive tasks requiring directed/executive control of attention, which were utilized in the reviewed studies, since very few studies used these tasks and those that did found no clear effect of the natural environment exposures.

## General Discussion

In this paper we analyzed the effects of nature vs. urban environment interactions on directed attention, measured by the BDS task, across 12 experimental studies. Furthermore, we assessed how affect and the order of environmental conditions influenced the effects of environment on cognitive performance.

Across the studies presented and analyzed in this paper, cognitive performance was found to improve significantly more after nature interactions compared to urban interactions. Overall, BDS performance improved on average by 0.75 (*SD* = 2.11) points from pre- to post-nature interactions and 0.29 (*SD* = 2.19) BDS points from pre- to post urban environment interactions [see Supplementary Table 13 in Appendix B].

However, we found a strong interaction between time (pre- vs. post environment exposure), environment type (nature vs. urban), and the session *order* of environmental conditions for studies with environment as a within-participants factor. Specifically, cognitive improvements were generally larger in first sessions than second sessions across different environmental conditions (e.g., nature, urban, and other control conditions), indicating a general practice effect in first testing sessions that is largely unrelated to the environmental exposure. Specifically, in first *sessions*, only a small advantage was seen for nature interactions (*M* = 0.97, *SD* = 2.06, BDS point improvement), compared to urban interactions (*M* = 0.73, *SD* = 2.35). On the other hand, *in the second testing sessions* (without the initial practice effects), the effects of the different environment conditions on cognitive performance were magnified, whereby performance continued to improve after the nature interactions by 0.51 (*SD* = 2.15) BDS points on average, while among those who interacted with urban environments in second sessions there was instead a trending decline in performance by −0.23 (*SD* = 1.87, *p* = 0.097) BDS points on average (see Table 3). These results help to clarify which effects are attributable to environmental conditions and demonstrate that initial practice effects must be parceled out to isolate cognitive effects from environmental interactions that are not confounded by practice effects (see Diagram 1). While these results show that the nature interactions tended to improve performance significantly, they also show that urban interactions may even cause declines in directed attention performance in some cases, as indicated by the decline in performance after interacting with urban environments in second testing sessions.

With regards to affect, although nature interactions generally have a positive effect on affect when compared to urban interactions, the differential effects of environmental exposure on directed attention (as measured by BDS) could not be explained by changes in affect. Specifically, in the nature condition, devoid of practice effects on the BDS task, changes in positive affect could only explain 2.2% of the variability in BDS change from pre- to post- the nature interaction. These results thus imply that the positive effects of nature interactions on executive/directed attention and on self-perceived affect are largely independent. However, it is also possible that limitations in the measurement of affect could prevent the detection of existing covariation in affect and cognitive performance changes resulting from environmental influences. Such limitations could be the result of the nature of affect rating scales (with possible floor vs. ceiling effects for negative vs. positive items), as well as individual variability in perceptiveness/awareness of one's own affective state and changes in affective state.

The boost in cognitive performance following nature interactions may be caused by a replenishment of cognitive resources mediated by a rest of executive cognitive-control processes, as suggested by ART. That is, natural environments and their stimuli tend to be “softly fascinating” and activate involuntary attention, while not demanding any particular actions by the observer that tap effortful directed attention, thus resting effortful directed-attention. The characteristic perceptual features of natural stimuli, in terms of containing statistical fractal patterns, may contribute to this effect, since such patterns have been found to induce brain signals related to a wakefully relaxed state (Hägerhäll et al., 2015). Furthermore, the soft fascination that comes from perceiving natural stimuli may also facilitate a form of restful distraction from, and reduction in, other effortful cognitive processes that compete for attentional resources (and which can thereby have a negative impact on executive cognitive performance). Examples of these phenomena are the negative effects of proactive interference (whereby recently activated items in working memory interfere with current to-be-remembered items and task goals), and ruminative thinking, which similarly can interfere with cognitive task performance (Brinker et al., 2013). Interacting with natural environments may instead facilitate a “stilling of the mind” (Stenfors et al., 2018). Evidence in support of the hypothesis that interactions with nature may facilitate a distraction from other cognitions, and as such a stilling of the mind, were reported by Bratman et al. (2015b) who found that ruminative thought processes were reduced after a nature walk but not an urban walk.

The nature-related performance boost could also stem from an increased motivational state, modulating the deployment of neural resources and functioning of neural networks involved in executive cognitive-control processes. It has also been suggested that natural environments and stimuli tend to have a positive effect on well-being because these are the environments in which humans evolved, and they contain the resources that enable human survival (e.g., food, water, raw materials, shelter; Appleton, 1975/1996; Wilson, 1984; Ulrich, 1991; McMahan and Estes, 2015; Dosen and Ostwald, 2016). According to this reasoning, environments that signal that resources for survival are in abundance (such as vegetation, water, raw material, shelter, biodiversity), while threats to survival are absent or low, should have the most positive effects and be the most preferred, for which there is some support (McMahan and Estes, 2015; Wyles et al., 2017). This way, environmental stimuli are plausibly linked to the up- and down-regulation of motivational systems (McMahan and Estes, 2015) which signal reward and modulate approach behaviors. These systems include e.g., parts of the basal ganglia, ventral tegmental area, and substantia nigra with dopaminergic projections to wide areas of the cerebral cortex and subcortical areas where dopamine signaling regulate neural activity (Botvinick and Braver, 2015). Importantly, these motivational systems are intimately related to and subserve the regulation of executive cognitive-control processes and associated neural networks, as well as the experience of effort and cognitive fatigue (Botvinick and Braver, 2015). If one of the underlying effects of natural stimuli is to induce a motivational boost, this would also be a plausible explanation for the positive effects that can occur from very brief exposures to natural stimuli, such as viewing images of nature for a short duration. Future research should investigate such possible underlying mechanisms of the observed behavioral effects of interactions with nature. A summary of the observed effects of environment exposure and test order on cognitive performance changes, and the potential mechanisms discussed above, are illustrated in Diagram 1.

### The Possible Role of Exposure Type and Duration for Restorative Effects

Generally, among all the studies by Berman et al. presented and analyzed in this paper, the effects of virtual reality nature and picture exposures were smaller, compared to walks in real nature as well as nature exposure via a video. It is noteworthy that the walks in real environments at the University of Chicago, which were shorter and less contrasted in terms of nature and urban built elements than those at the University of Michigan, showed smaller effects. This is understandable and suggests that the extent of naturalness vs. urban-ness plays a role for the positive effects on cognitive performance that are gained. This is in line with other recent research findings of dose-response effects on stress recovery from experimentally controlled environments with different amounts of natural elements (Jiang et al., 2016), and that longer and more frequent visits to green spaces among urban dwellers was associated with reduced risks of depression (Shanahan et al., 2016). However, not all data are consistent on this point. In a different healthy sample (screened to be free from mental illness), the effects of the same nature walk on BDS performance was not superior compared to the urban walk at the University of Michigan. Moreover, participants showed significant improvements across both nature and urban conditions in both the first and second sessions. It is possible that this sample consisted of individuals who were more robust to fatigue effects, were less affected by the environment, and had a high cognitive learning capacity. At the other end of the fatigue spectrum, the results for the participant sample who were clinically diagnosed with major depression exhibited the largest effect of the nature walk compared to the urban walk at University of Michigan. These effects were due to clear improvements in the nature condition, while notable deterioration in BDS task performance occurred after the urban condition, which is especially evident in the second sessions where the initial practice effects were not present.

### Strengths and Limitations

The present paper reported the results of all experiments conducted by Berman and colleagues, which met the inclusion criteria, with the purposes of analyzing and delineating the effects of session order as well as affect (data which are not available for other studies) from the effects of environmental exposures on BDS. A strength of this paper is the large amount of data that is analyzed, and the absence of publication bias for the individual experimental studies (non-significant results are less prevalent in published samples, but are included here). Another strength of this multiple-experiments study was the use of the same cognitive test measure across all studies, allowing for both comparability between studies and a more robust analysis across different studies of the effects of nature vs. control exposures on executive cognitive performance as measured by the BDS task.

When reviewing the results of other studies in the literature, the qualitative, and quantitative summaries were limited to the information presented in those study reports. Incomplete reporting of descriptive statistics for each experimental condition and dependent variables, in some studies, limited the review of and comparisons to other's findings in studies testing the effects of nature and control exposures on BDS performance.

## Conclusions

Pooled data-analyses were performed on a total of 528 participants across 12 studies with different types of interactions with nature vs. urban environments. Significant environment × time interactions were found whereby BDS performance improved more after nature compared to urban environmental interactions. Importantly, this effect was magnified after parceling out initial practice effects on the BDS task. In this case, BDS performance instead declined after urban environment interactions in some studies, indicating a fatigue effect, while BDS performance continued to improve after nature interactions. Furthermore, the cognitive performance improvements after nature interactions were found to be largely independent of changes in positive and negative affect. These results suggest that the mechanisms through which nature interactions alter cognitive performance vs. affect may differ and be independent.

Other studies in the literature examining the effects of nature vs. urban or other control environment interactions on BDS performance showed mixed results where some found clear practice effects that could have overshadowed the detection of any environmental effect. Effects of order and practice should thus be handled carefully in future studies to obtain more accurate estimates of the effects that different environmental interactions have on cognitive performance.

## Ethics Statement

All studies were approved by and carried out in accordance with the recommendations of the Institutional Review Boards for psychology at the University of Chicago, the University of Michigan, and the University of British Columbia. All subjects gave written informed consent in accordance with the Declaration of Helsinki.

## Author Contributions

Conception and design of the present paper: CS and MB. Data-base construction, data-analyses, and writing of the manuscript: CS. Conception, design, and data collection in individual experimental studies: MB, SV, KES, FM, KELS, GN, SB, JE, OK, and JJ. Critical review of the manuscript: MB, SV, KES, FM, KELS, GN, SB, JE, OK, JJ, and CS.

### Conflict of Interest Statement

The authors declare that the research was conducted in the absence of any commercial or financial relationships that could be construed as a potential conflict of interest.
