# Corrigendum: Positive Effects of Nature on Cognitive Performance Across Multiple Experiments: Test Order but Not Affect Modulates the Cognitive Effects

**DOI:** 10.3389/fpsyg.2019.02242

**Published:** 2019-10-17

**Authors:** Cecilia U. D. Stenfors, Stephen C. Van Hedger, Kathryn E. Schertz, Francisco A. C. Meyer, Karen E. L. Smith, Greg J. Norman, Stefan C. Bourrier, James T. Enns, Omid Kardan, John Jonides, Marc G. Berman

**Affiliations:** ^1^Department of Psychology, University of Chicago, Chicago, IL, United States; ^2^Department of Neurobiology, Care Science and Society, Aging Research Center, Karolinska Institute, Stockholm, Sweden; ^3^Department of Psychology, Stockholm University, Stockholm, Sweden; ^4^Department of Psychology, University of British Columbia, Vancouver, BC, Canada; ^5^Department of Psychology, University of Michigan, Ann Arbor, MI, United States

**Keywords:** cognitive restoration, cognitive performance, directed attention, nature, environment, affect, practice effects, order effects

In the original article, there was a mistake in Diagram 1 as published. Some information for the “total effects” in the diagram undertext/description for part E and F was incorrect (specifically the standard errors, confidence intervals, and *t*-values for the “total effect” estimate) due to a programming error in the MEMORE-macro v.2 for SPSS, which was used for the supplementary mediational path analyses, illustrated in Diagrams 1E,F (and presented in Appendix C).

**Diagram 1:**
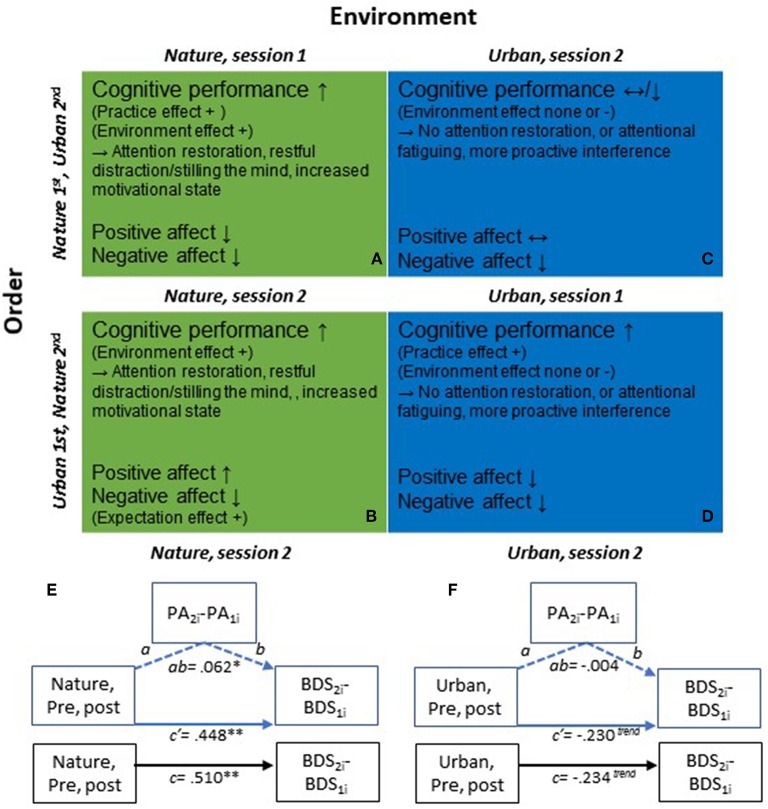
**(A–D)** Illustration of general effects and potential mechanisms of action on cognitive performance and affect from pre- to post nature vs. urban environment interactions, and the order of participation in the environment conditions. **(A)** Nature environment interaction, Nature condition is tested in session 1; **(B)** Nature environment interaction, Nature condition is tested in session 2; **(C)** Urban environment interaction, Urban condition is tested in session 2; **(D)** Urban environment interaction, Urban condition is tested in session 1. Depending on the order in which individuals participate in the nature and urban conditions, the changes observed on cognitive performance and affect also differ. In first test sessions **(A,D)**, there are clear practice effects on cognitive performance in addition to the effects that are caused by the environment condition *per se*. Second test sessions **(B,C)** are instead devoid of the initial performance improvement due to practice, and the cognitive performance improvements observed constitute more clean effects of the environment conditions. Thus, the second sessions provide a better evaluation of the effects of the environment conditions *per se* on cognitive performance. Changes in affect also differ depending on the order of environment conditions. That is, positive affect increased after nature interactions performed in the second but not first sessions, which could be due to different expectations on the second session, depending on the experience (environment condition) in the prior, first session. That is, if the urban condition was done first, the expectations on the second condition may be low and the actual experience after the environment interaction in session 2 may have exceeded expectations. **(E,F)** Mediational path analyses of total, direct & indirect effects on BDS performance, via positive affect (PA), following nature vs. urban environment interactions, in 2nd test sessions. **(E)** Nature condition, 2nd test sessions: Total effect, c: 0.510 (*SE: 0.154, t* = *3.320, df* = *194, 95% CI: 0.207, 0.813*); Direct effect, c′ : 0.448 (*SE: 0.155, t* = *2.885, df* = *192, 95% CI: 0.142, 0.755*); Indirect effect, ab: 0.062 (*SE: 0.038, 95% CI: 0.001, 0.145*). **(F)** Urban condition, 2nd test sessions: Total effect, *c*: −0.234 (*SE: 0.137, t* = −*1.714, df* = *187, 95% CI:* −*0.504, 0.035*); Direct effect, c′ : −0.230 (*SE: 0.138, t* = −*1.670, df* = *185, 95% CI:* −*0.503, 0.042*); Indirect effect, *ab*: −0.004 (*SE: 0.019, 95% CI:* −*0.050, 0.032*). ^**^p < *0.01*, ^*^p < *0.05*, ^*trend*^*p* < *0.10*.

The programming error pertained to the output for the standard errors of the “total effects” estimates, in the output of the MEMORE-macro v.2. The standard errors, confidence intervals, and *t*-values for the “total effect” estimate in the descriptions of part E and part F in Diagram 1 have now been corrected. Indication of the statistical significance level of the total effect in part E-F of the diagram artwork has also been corrected accordingly.

In the supplementary material, in **Appendix C**, supplementary mediational path analyses are presented on the total, direct & indirect effects on BDS performance, *via* positive affect (PA), following nature vs. urban environment interactions, in 1st vs. 2nd test sessions.

The standard errors, confidence intervals, and *t*-values for the total effects estimates only, were incorrect, due to a programming error pertaining to the standard errors, in the output of the MEMORE-macro v.2, which was used to perform the supplementary mediational path analyses presented in **Appendix C**. **Appendix C** has now been corrected.

The authors apologize for these errors and state that they do not change the scientific conclusions of the article in any way. The original article has been updated.

