# Supplementary material for: Positive Effects of Nature on Cognitive Performance Across Multiple Experiments: Test Order but Not Affect Modulates the Cognitive Effects
Source: Front Psychol. 2019 Jul 3;10:1413. doi: 10.3389/fpsyg.2019.01413 (PMC6616085; doi:10.3389/fpsyg.2019.01413)
Supplement: Supplementary file 1 [file Table_1.pdf]

Appendix A

Contents

1. Supplementary information on procedures and methods for new and previously unpublished studies that are reported in the paper
2. Supplementary information on statistical analyses and results presented in the manuscript

1. Procedures and methods for all new and previously unpublished studies that are reported in the paper

Walk study, University of Chicago, 2016:

University students performed the BDS task and the PANAS, administered via E-prime, before and after a 20 min walk on campus with more versus less natural stimuli (and less versus more urban/built features in the environment). In the BDS task, a string of 3-9 digits was presented auditorily in each trial. String lengths were presented in random order, with 2 trials of each string length. Effects of the respective environment conditions were tested in a within-subjects design, in which participants were randomly allocated to the order of conditions which were tested one week apart. In the more natural environment, participants walked within the main quadrangles of the University of Chicago campus, consisting of more or less open spaces with an abundance of trees, grass, vines on the walls of buildings, other kinds of vegetation and a pond. In the urban condition, participants walked the same distance along the streets outside the main quadrangles of the University of Chicago, characterized by a greater density of buildings (buildings along both sides of the roads), traffic, and less vegetation.

Virtual Reality 1 (VR 1), University of Chicago, 2016:

The study employed a RCrT design with the environment conditions tested one week apart. University students performed the BDS task and the PANAS, administered via E-prime, before and after viewing/experiencing a nature or an urban/built environment in VR for 8 minutes (see Diagram 1). In the BDS task, a string of 3-11 digits was presented auditorily in each trial. String lengths were presented in random order, with 2 trials of each string length. The virtual reality environments were created using Unity version 5.2.2, and compiled for Windows computers. These environments were designed for display on an Oculus Rift Developer Kit 2 (DK2) virtual reality head mounted device. Elements for each environment were drawn from Unity Store assets. The practice environment was adapted from the Unity Test Scene from the v5.2.2 standard assets (Unity, 2015); the nature environment was generated using the TerrainComposer asset v1.96 (Doldersum, 2015); the urban environment was adapted from the Modern City Pack environment November 2015 release (noirfx, 2015). Irrespective of the environment, the same back-end scripts were used. Participants' physical position in the map was fixed, in order to minimize the risk for nausea. However, participants were allowed to look around a 360 view by moving their headset. Participants were also able to teleport between 4 different locations in each environment at will, by pressing the A/B/X/Y

buttons on an Xbox 360 controller (see Diagram 1) for Windows (Microsoft, 2004). The number of teleports was tracked and the locations to which participants moved; in addition, a screen recorder app was used to record what participants were seeing while they were going through the procedure using a screen recorder app.

Virtual reality 2 (VR 2), University of Chicago, 2016:

Environment conditions were similar as in VR 1, but in VR 2 the effects of the environment conditions were tested in an RCT design. University student participants came in to the lab at 2 occasions. In session one, participants were exposed to a control environment (a moon environment) and half of the participants experienced this environment in virtual reality to provide an opportunity to habituate to the VR experience (including wearing the VR goggles and changing perspective/scene), while the other half just watched the same environment on a PC screen without the opportunity to habituate to the VR experience before the next session. In the second session, one week later, participants were randomly allocated to experience either a natural or an urban/built environment in VR. The BDS task and the PANAS were administered before and after the environment exposures in both session 1 and 2. In the BDS task, a string of 3-11 digits was presented auditorily in each trial. String lengths were presented in random order, with 2 trials of each string length.

The moon environment used in the first sessions was adapted from the Moon Environment package (Svchost74, 2015) and was created by similar procedures as the nature and urban environments.

Diagram A1. Illustrations of the nature and urban virtual reality conditions:

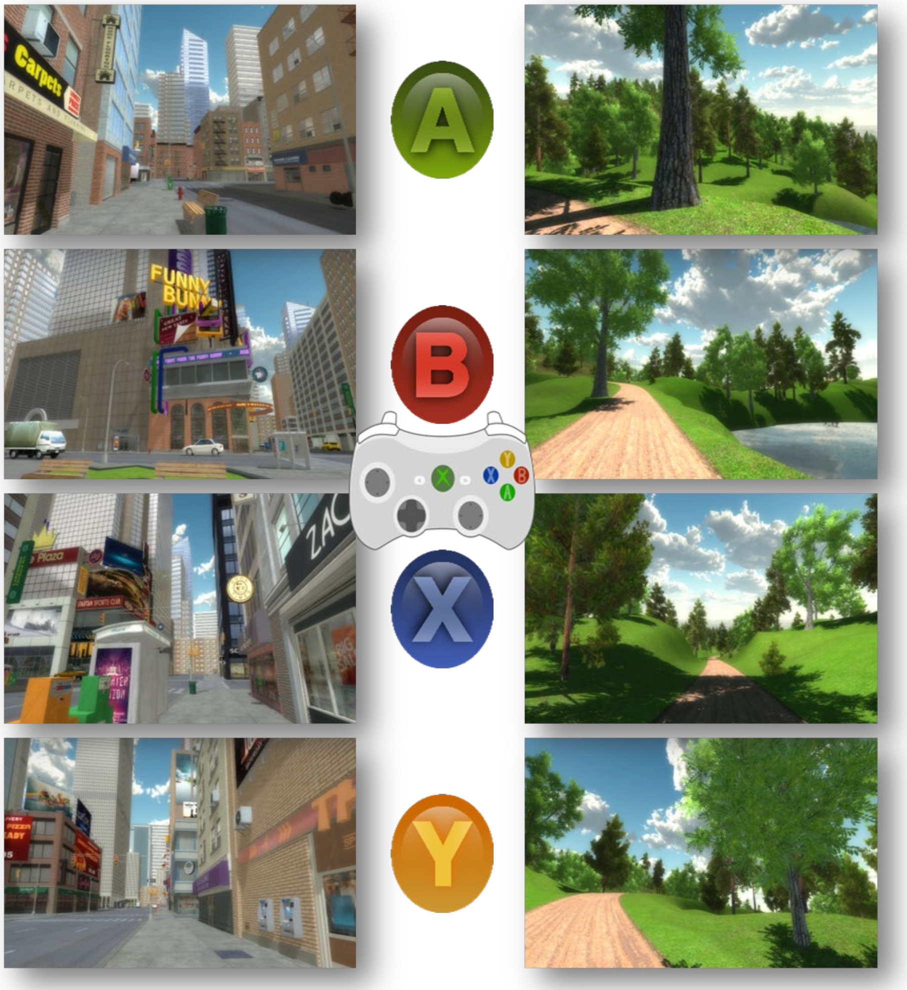

Composite study - visual environments, University of Chicago, 2016 (Van Hedger et al. unpublished data):

Participants from the University of Chicago completed a PANAS, BDS, and dual n-back task before and after viewing nature or urban pictures (between-participants), using an RCT design. In the BDS task, a string of 3-9 digits was presented in each trial. String lengths were presented in random order, with 2 trials of each string length. For the environment conditions, a total of 200 images were used. Half of the images were representative of nature (e.g., forest, desert, and ocean scenes), while the other half of the images were representative of urban environments, all containing some sort of built structure outdoors (e.g., single family home, office building, skyscraper, city skyline). No image contained any people or animals. These images were preselected from a larger database (Xiao, Ehinger et al. 2016) that had been rated on naturalness (Kardan, Demiralp et al. 2015). The naturalness ratings were completely non-overlapping, in that the lowest rated nature image was rated as more natural than the highest-rated urban image. Each image was 1200x900 pixels. The experiment was completed in a single session lasting approximately 60 minutes.

Picture study, University of Chicago & Picture study, University of Michigan, 2015:

University students performed the BDS task and the PANAS, administered via E-prime, before and after viewing 50 images of either natural (scenery of Nova Scotia) or urban environments (from Ann Arbor, Detroit, and Chicago), in a RCrT design with the different environment conditions tested one week apart.

The environment conditions and picture stimuli in the nature versus urban conditions were the same as in experiment 2 in Berman et al. 2008 (Berman, Jonides et al. 2008). Each picture viewing condition lasted approximately 10 min, during which participants rated on a scale of 1 to 3 how much they liked each picture. Pictures were displayed for 7 s, followed by a rating interval that lasted until the participant responded.

Healthy sample, University of Michigan, 2011:

The study employed a RCrT design with the environment conditions tested one week apart. Participants were university students without any physical or mental health conditions (depression and anxiety in particular). Participants performed the BDS task and the PANAS, before and after a 50-55 min walk in a natural or urban environment. In the BDS task, a string of 3-9 digits was presented in each trial, with 2 trials at each string length. The nature walk took place in Nichols arboretum in Ann Arbor, and the urban walk of the same length took pace along a main street in the city of Ann Arbor with a high density of buildings, traffic, and little vegetation.

Picture dose response, University of Michigan, 2009:

University students performed the BDS task and the PANAS, administered via E-prime, before and after viewing either 25, 50, or 100 images of natural environments and were tested on two occasions one week apart. Participants were randomly allocated to the picture dose. Because no differences in effects were seen between the different picture doses, data for all participants are presented and analyzed together. Although this study lacks a control group viewing a non-natural environment, this sample was included in this multiple experiments paper since one of the main aims of this paper was to evaluate the effect of order, or practice, and delineate this from the effects of the environment per se. Because participants were tested on two consecutive occasions in this study, this provides a relevant test of the practice effect versus the effect which may be due to the environment exposure per se.

2. Supplementary information on the statistical analyses and results presented in the manuscript sections 3.1. (incl. table 7) and section 3.3. (incl. table 11)

2.1. Supplementary information on the results presented in the manuscript in table 7

- Descriptions, syntaxes and model results from general linear models computed in the Linear Mixed Models (LMM) procedure in SPSS, testing the effects of time (pre, post) by environment (nature, urban) by order (nature 1st vs urban 1st) on BDS performance, while adjusting for affect (positive vs. negative affect) at each time point, including studies with environment as a within-subjects factor.

General linear model analyses testing the (fixed) effects of the factors time, environment and order (testing the full factorial model) on BDS performance, while also including positive affect (PA), vs. negative affect (NA), as time-varying covariates (i.e. affect was measured at the same time points as the BDS task), were computed in the LMM procedure in SPSS 25. The LMM procedure was used for this precisely because it allows the inclusion of time-varying covariates when testing the factorial model as described. Maximum likelihood estimation was used and unstructured covariance type for the repeated factors (time and environment). In these models, including affect as time-varying covariates, the effects of the factors time, environment and order on BDS performance were thus adjusted for the effects of affect levels on BDS performance at each measurement point. By comparing the estimated effects of the factors on BDS without versus with adjustments for affect, one can evaluate if the effects of the factors on BDS can be explained (or mediated) by effects on affect.
In the analyses, the following factors were analysed: time ("time_pre_post", pre was coded as 1, post was coded as 2); environment condition ("Env", nature was coded as 1, urban was coded as 2); and order ("Order", nature condition 1st was coded as 1, urban condition 1st was coded as 2). BDS (BDS_score, continuous variable) was the dependent variable. PA and NA were continuous variables measured at each and the same time points as BDS and were included in the analyses as time-varying covariates

Fixed effects models computed in the LMM procedure are general linear models similar to the computations in the GLM procedure (i.e. simple ANOVAs and repeated measures ANOVAs). A fitted fixed effects model has the form $y = X\beta + \varepsilon$, where y is a vector of responses, and X is the
fixed-effects design matrix, β is a vector of fixed-effects parameters and ε is a vector of residual errors. It is assumed that ε is distributed as $R = \sigma^2 I$, where R is an unknown covariance matrix. (SPSS Technical Report: http://www.spss.ch/upload/1126184451_Linear%20Mixed%20Effects%20Modeling%20in%20SPSS.pdf)

2.1.1. Time x environment x order effects on BDS with adjustment for PA as a time-varying covariate

SPSS syntax

```
MIXED BDS_score BY time_pre_post Order WITH PA
  /CRITERIA=CIN(95) MXITER(100) MXSTEP(10) SCORING(1)
SINGULAR(0.000000000001) HCONVERGE(0,
    ABSOLUTE) LCONVERGE(0, ABSOLUTE) PCONVERGE(0.000001, ABSOLUTE)
  /FIXED=time_pre_post Order time_pre_post*Env time_pre_post*Order
    Env*Order time_pre_post*Env*Order PA | SSTYPE(3)
  /METHOD=ML
  /PRINT=CORB COVB DESCRIPTIVES G  LMATRIX R SOLUTION TESTCOV
  /REPEATED=time_pre_post*Env | SUBJECT(Meta_ID) COVTYPE(UN).
```

Supplementary table 7.1. Fixed effects and parameter estimates for the factorial model time x environment x order effects on BDS, with PA as a time-varying covariate

Type III Tests of Fixed Effects[a]

Source	Numerator df	Denominator df	F	Sig.

Intercept	1	957.885	1271.581	.000
time_pre_post	1	299.778	47.996	.000
Env	1	301.913	2.635	.106
Order	1	302.157	2.705	.101
time_pre_post * Env	1	304.091	5.731	.017
time_pre_post * Order	1	300.274	3.668	.056
Env * Order	1	316.371	64.152	.000
time_pre_post * Env * Order	1	304.323	18.843	.000
PA	1	975.600	2.726	.099

Estimates of Fixed Effects[a]

Parameter	Estimate	Std. Error	df	t	Sig.	95% Confidence Interval	
						Lower Bound	Upper Bound
Intercept	9.074235	.302498	715.471	29.998	.000	8.480345	9.668124
[time_pre_post=1]	-.906507	.163435	303.212	-5.547	.000	-1.228117	-.584898
[time_pre_post=2]	0[b]	0	.	.	.	.	.
[Env=1]	.824845	.188513	299.180	4.376	.000	.453866	1.195823
[Env=2]	0[b]	0	.	.	.	.	.
[Order =1]	-.124720	.297682	303.352	-.419	.676	-.710503	.461063
[Order=2]	0[b]	0	.	.	.	.	.
[time_pre_post=1] * [Env=1]	.325970	.235208	305.729	1.386	.167	-.136860	.788801
[time_pre_post=1] * [Env=2]	0[b]	0	.	.	.	.	.
[time_pre_post=2] * [Env=1]	0[b]	0	.	.	.	.	.
[time_pre_post=2] * [Env=2]	0[b]	0	.	.	.	.	.
[time_pre_post=1] * [Order=1]	1.048609	.232320	301.987	4.514	.000	.591439	1.505779
[time_pre_post=1] * [Order=2]	0[b]	0	.	.	.	.	.
[time_pre_post=2] * [Order=1]	0[b]	0	.	.	.	.	.
[time_pre_post=2] * [Order=2]	0[b]	0	.	.	.	.	.
[Env=1] * [Order=1]	-.920585	.270030	304.145	-3.409	.001	-1.451947	-.389222
[Env=1] * [Order=2]	0[b]	0	.	.	.	.	.
[Env=2] * [Order=1]	0[b]	0	.	.	.	.	.
[Env=2] * [Order=2]	0[b]	0	.	.	.	.	.
[time_pre_post=1] * [Env=1] * [Order=1]	-1.452741	.334667	304.323	-4.341	.000	-2.111296	-.794187

[time_pre_post=1] * [Env=1] * [Order=2]	0[b]	0	.	.	.	.	.
[time_pre_post=1] * [Env=2] * [Order=1]	0[b]	0	.	.	.	.	.
[time_pre_post=1] * [Env=2] * [Order=2]	0[b]	0	.	.	.	.	.
[time_pre_post=2] * [Env=1] * [Order=1]	0[b]	0	.	.	.	.	.
[time_pre_post=2] * [Env=1] * [Order=2]	0[b]	0	.	.	.	.	.
[time_pre_post=2] * [Env=2] * [Order=1]	0[b]	0	.	.	.	.	.
[time_pre_post=2] * [Env=2] * [Order=2]	0[b]	0	.	.	.	.	.
PA	.142003	.086008	975.600	1.651	.099	-.026780	.310785

a. Dependent Variable: BDS score.

b. This parameter is set to zero because it is redundant.

2.1.2. Time x environment x order effects on BDS with adjustment for PA as a time-varying covariate

SPSS syntax

```
MIXED BDS_score BY time_pre_post Order WITH NA
  /CRITERIA=CIN(95) MXITER(100) MXSTEP(10) SCORING(1)
SINGULAR(0.000000000001) HCONVERGE(0,
    ABSOLUTE) LCONVERGE(0, ABSOLUTE) PCONVERGE(0.000001, ABSOLUTE)
  /FIXED=time_pre_post Order time_pre_post*Env time_pre_post*Order
    Env*Order time_pre_post*Env*Order NA | SSTYPE(3)
  /METHOD=ML
  /PRINT=CORB COVB DESCRIPTIVES G  LMATRIX R SOLUTION TESTCOV
  /REPEATED=time_pre_post*Env | SUBJECT(Meta_ID) COVTYPE(UN).
```

Supplementary table 7.2. Fixed effects and parameter estimates for the factorial model time x environment x order effects on BDS, with NA as a time-varying covariate

Type III Tests of Fixed Effects[a]

Source	Numerator df	Denominator df	F	Sig.
Intercept	1	863.728	1826.778	.000
time_pre_post	1	302.952	39.289	.000
Env	1	292.778	2.402	.122
Order	1	291.737	1.842	.176
time_pre_post * Env	1	291.335	5.822	.016
time_pre_post * Order	1	289.657	3.605	.059
Env * Order	1	291.268	56.301	.000
time_pre_post * Env * Order	1	291.029	17.468	.000

NA	1	974.437	1.477	.225

Estimates of Fixed Effects[a]

Parameter	Estimate	Std. Error	df	t	Sig.	95% Confidence Interval Lower Bound	95% Confidence Interval Upper Bound
Intercept	9.667287	.278716	599.572	34.685	.000	9.119908	10.214666
[time_pre_post=1]	-.867227	.166977	294.956	-5.194	.000	-1.195844	-.538611
[time_pre_post=2]	0[b]	0	.	.	.	.	.
[Env=1]	.784484	.191858	291.085	4.089	.000	.406881	1.162088
[Env=2]	0[b]	0	.	.	.	.	.
[Order=1]	-.102497	.303143	291.504	-.338	.736	-.699123	.494130
[Order=2]	0[b]	0	.	.	.	.	.
[time_pre_post=1] * [Env=1]	.303343	.239851	289.497	1.265	.207	-.168730	.775415
[time_pre_post=1] * [Env=2]	0[b]	0	.	.	.	.	.
[time_pre_post=2] * [Env=1]	0[b]	0	.	.	.	.	.
[time_pre_post=2] * [Env=2]	0[b]	0	.	.	.	.	.
[time_pre_post=1] * [Order=1]	1.045583	.237449	291.360	4.403	.000	.578250	1.512916
[time_pre_post=1] * [Order=2]	0[b]	0	.	.	.	.	.
[time_pre_post=2] * [Order=1]	0[b]	0	.	.	.	.	.
[time_pre_post=2] * [Order=2]	0[b]	0	.	.	.	.	.
[Env=1] * [Order=1]	-.833454	.274470	290.590	-3.037	.003	-1.373655	-.293253
[Env=1] * [Order=2]	0[b]	0	.	.	.	.	.
[Env=2] * [Order=1]	0[b]	0	.	.	.	.	.
[Env=2] * [Order=2]	0[b]	0	.	.	.	.	.
[time_pre_post=1] * [Env=1] * [Order=1]	-1.435750	.343523	291.029	-4.179	.000	-2.111855	-.759646
[time_pre_post=1] * [Env=1] * [Order=2]	0[b]	0	.	.	.	.	.
[time_pre_post=1] * [Env=2] * [Order=1]	0[b]	0	.	.	.	.	.
[time_pre_post=1] * [Env=2] * [Order=2]	0[b]	0	.	.	.	.	.
[time_pre_post=2] * [Env=1] * [Order=1]	0[b]	0	.	.	.	.	.

[time_pre_post=2] * [Env=1] * [Order=2]	0[b]	0	.	.	.	.	.
[time_pre_post=2] * [Env=2] * [Order=1]	0[b]	0	.	.	.	.	.
[time_pre_post=2] * [Env=2] * [Order=2]	0[b]	0	.	.	.	.	.
NA	-.152017	.125080	974.437	-1.215	.225	-.397475	.093441

a. Dependent Variable: BDS score.

b. This parameter is set to zero because it is redundant.

2.2. Supplementary information on the results presented in the manuscript in section 3.1.3., including table 11

Due to the marked effects of the order of the environment conditions on BDS performance changes, analyses were computed after stratifying by session order (1st test sessions vs 2nd test sessions), including all samples which tested environment as a within- or a between-subjects factor. In these analyses, the environment condition is a therefore a between-subjects factor. That is, pre- to post changes in BDS after nature and urban conditions which were tested in the 1st session were compared in one analysis (session 1), and nature and urban conditions which were tested in the 2nd session were compared in another analysis (session 2). General linear model analyses were computed in the LMM procedure in SPSS 25, testing the (fixed) effects of the factors time and environment on BDS performance, and testing the same models while also including positive affect (PA), vs. negative affect (NA), as time-varying covariates (i.e. affect was measured at the same time points as the BDS task). Maximum likelihood model estimation was used and unstructured covariance type for the repeated factor (time).
In the analyses, the following factors were analysed: time ("time_pre_post", pre was coded as 1, post was coded as 2); environment condition ("Env", nature was coded as 1, urban was coded as 2). BDS (BDS_score, continuous variable) was the dependent variable. PA and NA were continuous variables measured at each and the same time points as BDS and were included as time-varying covariates in the models adjusting for PA vs. NA.

2.2.1. Time x environment effects on BDS

- Syntax and model results from the general linear model testing the effects of time by environment on BDS performance, stratified by session order (1st test sessions vs 2nd test sessions), including studies which tested environment as a within- or a between-subjects factor.

SPSS syntax

```
MIXED BDS_score BY time_pre_post Env
  /CRITERIA=CIN(95) MXITER(100) MXSTEP(10) SCORING(1)
SINGULAR(0.000000000001) HCONVERGE(0,
    ABSOLUTE) LCONVERGE(0, ABSOLUTE) PCONVERGE(0.000001, ABSOLUTE)
  /FIXED=time_pre_post Env time_pre_post*Env | SSTYPE(3)
  /METHOD=ML
  /PRINT=CORB COVB DESCRIPTIVES G  LMATRIX R SOLUTION TESTCOV
  /REPEATED=time_pre_post | SUBJECT(Meta_ID) COVTYPE(UN).
```

Supplementary table 11.1. Fixed effects and parameter estimates for time x environment effects on BDS, for all samples stratified by 1st versus 2nd test sessions

Type III Tests of Fixed Effects[a]

Session order	Source	Numerator df	Denominator df	F	Sig.
Test session 1	Intercept	1	446.000	5894.507	.000
	time_pre_post	1	446.000	65.624	.000
	Env	1	446.000	.216	.642
	time_pre_post * Env	1	446.000	1.359	.244
Test session 2	Intercept	1	384.000	5850.396	.000
	time_pre_post	1	384.000	1.900	.169
	Env	1	384.000	3.297	.070
	time_pre_post * Env	1	384.000	12.936	.000

Estimates of Fixed Effects[a]

Session order	Parameter	Estimate	Std. Error	df	t	Sig.	95% Confidence Interval Lower Bound	95% Confidence Interval Upper Bound
Test session 1	Intercept	9.138274	.178280	446	51.258	.000	8.787902	9.488647
	[time_pre_post=1]	-.724558	.146758	446.000	-4.937	.000	-1.012981	-.436134
	[time_pre_post=2]	0[b]	0	.	.	.	.	.
	[Env=1]	.228771	.253839	446	.901	.368	-.270098	.727640
	[Env=2]	0[b]	0	.	.	.	.	.
	[time_pre_post=1] * [Env=1]	-.243624	.208958	446.000	-1.166	.244	-.654289	.167040
	[time_pre_post=1] * [Env=2]	0[b]	0	.	.	.	.	.
	[time_pre_post=2] * [Env=1]	0[b]	0	.	.	.	.	.
	[time_pre_post=2] * [Env=2]	0[b]	0	.	.	.	.	.
Test session 2	Intercept	9.396825	.194031	384.000	48.430	.000	9.015330	9.778321
	[time_pre_post=1]	.227513	.146173	384.000	1.556	.120	-.059886	.514913
	[time_pre_post=2]	0[b]	0	.	.	.	.	.
	[Env=1]	.831380	.272282	384.000	3.053	.002	.296030	1.366730
	[Env=2]	0[b]	0	.	.	.	.	.
	[time_pre_post=1] * [Env=1]	-.737770	.205123	384.000	-3.597	.000	-1.141075	-.334464
	[time_pre_post=1] * [Env=2]	0[b]	0	.	.	.	.	.
	[time_pre_post=2] * [Env=1]	0[b]	0	.	.	.	.	.

	[time_pre_post=2] * [Env=2]	0[b]	0	.	.	.	.	.
a. Dependent Variable: BDS score.								
b. This parameter is set to zero because it is redundant.								
Env=1=Nature; Env=2=Urban.								

2.2.2. Time x environment effects on BDS, with PA as a time-varying covariate

- Syntax and results from the general linear model testing the effects of time by environment on BDS performance, with positive affect (PA) as a time-varying covariate, to evaluate if effects on PA may explain effects of time by environment on BDS. The analyses include studies with environment as a within and a between-subjects factor and are stratified by session order (1st test sessions vs 2nd test sessions).
In this model, the following factors were analysed: time ("time", pre was coded as 1, post was coded as 2); environment condition ("Env", nature was coded as 1, urban was coded as 2). BDS (BDS_score, continuous variable) was the dependent variable. PA (measured at each and the same time points as BDS) was included as time-varying covariate.

SPSS syntax

```
MIXED BDS_score BY time_pre_post Env WITH PA
  /CRITERIA=CIN(95) MXITER(100) MXSTEP(10) SCORING(1)
SINGULAR(0.000000000001) HCONVERGE(0,
    ABSOLUTE) LCONVERGE(0, ABSOLUTE) PCONVERGE(0.000001, ABSOLUTE)
  /FIXED=time_pre_post Env time_pre_post*Env PA | SSTYPE(3)
  /METHOD=ML
  /PRINT=CORB COVB DESCRIPTIVES G  LMATRIX R SOLUTION TESTCOV
  /REPEATED=time_pre_post | SUBJECT(Meta_ID) COVTYPE(UN).
```

Supplementary table 11.2. Fixed effects F statistics and parameter estimates for time x environment effects on BDS, with PA as a time-varying covariate, stratified by session order.

Type III Tests of Fixed Effects[a]

Session order	Source	Numerator df	Denominator df	F	Sig.
Test session 1	Intercept	1	763.730	760.976	.000
	time_pre_post	1	388.360	62.501	.000
	Env	1	384.750	.109	.741
	time_pre_post * Env	1	382.138	.225	.635
	PA	1	747.858	.943	.332
Test session 2	Intercept	1	755.118	916.810	.000
	time_pre_post	1	383.769	1.637	.202
	Env	1	385.387	2.840	.093
	time_pre_post * Env	1	387.763	11.375	.001
	PA	1	755.770	4.784	.029

Estimates of Fixed Effects[a]

Session order	Parameter	Estimate		df	t	Sig.	95% Confidence Interval

			Std. Error				Lower Bound	Upper Bound
Test session 1	Intercept	9.051568	.339743	704.184	26.642	.000	8.384538	9.718598
	[time_pre_post=1]	-.828151	.155567	386.212	-5.323	.000	-1.134016	-.522287
	[time_pre_post=2]	0[b]	0	.	.	.	.	.
	[Env=1]	.131877	.265534	383.476	.497	.620	-.390209	.653962
	[Env=2]	0[b]	0	.	.	.	.	.
	[time_pre_post=1] * [Env=1]	-.104781	.220693	382.138	-.475	.635	-.538705	.329142
	[time_pre_post=1] * [Env=2]	0[b]	0	.	.	.	.	.
	[time_pre_post=2] * [Env=1]	0[b]	0	.	.	.	.	.
	[time_pre_post=2] * [Env=2]	0[b]	0	.	.	.	.	.
	PA	.106516	.109712	747.858	.971	.332	-.108865	.321896
Test session 2	Intercept	8.821682	.326527	680.159	27.017	.000	8.180560	9.462805
	[time_pre_post=1]	.216321	.146247	384.744	1.479	.140	-.071222	.503864
	[time_pre_post=2]	0[b]	0	.	.	.	.	.
	[Env=1]	.776377	.272840	387.237	2.846	.005	.239944	1.312810
	[Env=2]	0[b]	0	.	.	.	.	.
	[time_pre_post=1] * [Env=1]	-.694590	.205944	387.763	-3.373	.001	-1.099496	-.289683
	[time_pre_post=1] * [Env=2]	0[b]	0	.	.	.	.	.
	[time_pre_post=2] * [Env=1]	0[b]	0	.	.	.	.	.
	[time_pre_post=2] * [Env=2]	0[b]	0	.	.	.	.	.
	PA	.246546	.112715	755.770	2.187	.029	.025274	.467818

a. Dependent Variable: BDS score.

b. This parameter is set to zero because it is redundant.

Env=1=Nature; Env=2=Urban.

2.2.3. Time x environment effects on BDS, with NA as a time-varying covariate

- Syntax and results from general linear models testing the effects of time by environment on BDS performance, stratified by session order (1st test sessions vs 2nd test sessions), with negative affect (NA) as a time-varying covariate, to evaluate if effects on PA may explain effects of time by environment on BDS. The analyses include studies with environment as a within and a between-subjects factor.

In this model, the following factors were analysed: time (“time_pre_post”, pre was coded as 1, post was coded as 2); environment condition (“Env”, nature was coded as 1, urban was

coded as 2). BDS (BDS_score, continuous variable) was the dependent variable. NA (measured at each and the same time points as BDS) was included as time-varying covariate.

SPSS syntax

```
MIXED BDS_score BY time_pre_post Env WITH NA
  /CRITERIA=CIN(95) MXITER(100) MXSTEP(10) SCORING(1)
SINGULAR(0.000000000001) HCONVERGE(0,
    ABSOLUTE) LCONVERGE(0, ABSOLUTE) PCONVERGE(0.000001, ABSOLUTE)
  /FIXED=time_pre_post Env time_pre_post*Env NA | SSTYPE(3)
  /METHOD=ML
  /PRINT=CORB COVB DESCRIPTIVES G LMATRIX R SOLUTION TESTCOV
  /REPEATED=time_pre_post | SUBJECT(Meta_ID) COVTYPE(UN).
```

Supplementary table 11.3. Fixed effects F statistics and parameter estimates for time x environment effects on BDS, with PA as a time-varying covariate, stratified by session order.

Type III Tests of Fixed Effects[a]

Session order	Source	Numerator df	Denominator df	F	Sig.
Test session 1	Intercept	1	706.039	1170.667	.000
	time_pre_post	1	387.319	53.974	.000
	Env	1	375.567	.411	.522
	time_pre_post * Env	1	372.915	.214	.644
	NA	1	734.021	.484	.487
Test session 2	Intercept	1	720.576	1471.440	.000
	time_pre_post	1	384.143	.738	.391
	Env	1	374.195	2.213	.138
	time_pre_post * Env	1	373.927	12.099	.001
	NA	1	711.002	2.680	.102

Estimates of Fixed Effects[a]

Session order	Parameter	Estimate	Std. Error	df	t	Sig.	95% Confidence Interval Lower Bound	95% Confidence Interval Upper Bound
Test session 1	Intercept	9.498791	.301641	671.063	31.490	.000	8.906518	10.091064
	[time_pre_post=1]	-.792682	.158376	377.525	-5.005	.000	-1.104092	-.481272
	[time_pre_post=2]	0[b]	0	.	.	.	.	.
	[Env=1]	.207972	.269194	375.130	.773	.440	-.321348	.737291
	[Env=2]	0[b]	0	.	.	.	.	.
	[time_pre_post=1] * [Env=1]	-.104450	.225564	372.915	-.463	.644	-.547987	.339086
	[time_pre_post=1] * [Env=2]	0[b]	0	.	.	.	.	.
	[time_pre_post=2] * [Env=1]	0[b]	0	.	.	.	.	.
	[time_pre_post=2] * [Env=2]	0[b]	0	.	.	.	.	.

	NA	-.109489	.157298	734.021	-.696	.487	-.418297	.199320
Test session 2	Intercept	9.829567	.297395	680.481	33.052	.000	9.245644	10.413489
	[time_pre_post=1]	.273071	.150642	376.868	1.813	.071	-.023134	.569276
	[time_pre_post=2]	0[b]	0	.	.	.	.	.
	[Env=1]	.748088	.275344	373.999	2.717	.007	.206671	1.289505
	[Env=2]	0[b]	0	.	.	.	.	.
	[time_pre_post=1] * [Env=1]	-.729124	.209619	373.927	-3.478	.001	-1.141304	-.316945
	[time_pre_post=1] * [Env=2]	0[b]	0	.	.	.	.	.
	[time_pre_post=2] * [Env=1]	0[b]	0	.	.	.	.	.
	[time_pre_post=2] * [Env=2]	0[b]	0	.	.	.	.	.
	NA	-.265966	.162458	711.002	-1.637	.102	-.584921	.052989

a. Dependent Variable: BDS score.

b. This parameter is set to zero because it is redundant.

Env=1=Nature; Env=2=Urban.

2.2.4. Complementary post hoc analyses on the association between changes in BDS performance and changes in positive affect (PA) and negative affect (NA), in the nature versus urban condition, by session order

Supplementary table 11.4. Bivariate correlations between BDS change, PA change, NA change †, in the nature vs. urban conditions, by 1st vs. 2nd test sessions.

Session order	Environment condition	Estimate	PA change * BDS change	NA change * BDS change
1st test session	Nature	*r*	0.089	0.123
		p	0.228	0.098
		N	187	181
	Urban	*r*	0.073	-0.072
		p	0.310	0.321
		N	195	191
2nd test session	Nature	*r*	**.148***	-0.068
		p	**0.038**	0.350
		N	**195**	191
	Urban	*r*	0.020	-0.040
		p	0.790	0.593
		N	188	182

*. Correlation is significant at the 0.05 level (2-tailed).

† Change score= post score-pre score.

References

Berman, M. G., J. Jonides and S. Kaplan (2008). "The cognitive benefits of interacting with nature." Psychol Sci **19**(12): 1207-1212.

Kardan, O., E. Demiralp, M. C. Hout, M. R. Hunter, H. Karimi, T. Hanayik, G. Yourganov, J. Jonides and M. G. Berman (2015). "Is the preference of natural versus man-made scenes driven by bottom–up processing of the visual features of nature?" Frontiers in psychology **6**: 471.

Xiao, J., K. A. Ehinger, J. Hays, A. Torralba and A. Oliva (2016). "Sun database: Exploring a large collection of scene categories." International Journal of Computer Vision **119**(1): 3-22.
