# Supplementary material for: Positive Effects of Nature on Cognitive Performance Across Multiple Experiments: Test Order but Not Affect Modulates the Cognitive Effects
Source: Front Psychol. 2019 Jul 3;10:1413. doi: 10.3389/fpsyg.2019.01413 (PMC6616085; doi:10.3389/fpsyg.2019.01413)
Supplement: Supplementary file 2 [file Table_2.pdf]

Appendix B. Supplementary tables 12 and 13.

Table 12. Summary of the literature review. Studies testing effects of nature vs urban/control exposures on BDS test performance and other executive cognitive tests, using a randomized control- or a randomized crossover design.

Table 12. Summary of the literature review. Studies testing effects of nature vs urban/control exposures on BDS test performance and other executive cognitive tests, using a randomized control- or a randomized crossover design.

Studies using BDS[a]<br><br>Authors (year, experiment)	Design	N	Sample characteristics, Age, Gender etc	Type of Nature/Environment Intervention	Cognitive measures	Reported Statistical Values	BDS	FDS	FDS & BDS	ANT-E	NCPC	SART	TMTA	TMTB	Other
**Bodin et al**, (2003)	RCrT ⸙	12	12 runners, from runnning club. (50% female, mean age 37 (ffemales) 39.7 (meales) years. Sweden.	Running in natural (park) vs urban (city streets) setting for 60 min, 1 week apart.	**Combined Forward (FDS) & Backwards Digit Span (BDS), Symbol Digit Modalities Test.**	Sum of **BDS & FDS** reported. Time*Env interaction on FDS+BDS (men & women): F(1, 10)=0.92; p=0.36. Time*Env interaction on Symbol Digit Modalities test: F(1, 10)=0.02 , p=0.90.			n.s. †						Symbol digit modalities test: n.s.
**Bratman**, Daily, Levy, & Gross (2015).	RCT ⸙	60	Adults- univ students or people from the community (33 females, mean age 22.9 years). San Francisco Bay Area, USA.	50-minute walk in natural or urban environment (with 15-minute shuttle each way).	**BDS:** 3-9 digit strings (2 of each length). Test stops after 2 consecutive errors; DV=nr of correct sequences recalled. **Ospan, Attention Network task- Executive component (ANT-E), Change detection**	**BDS:** ANOVA results showed a main effect of time, F(1,58) = 8.40, p < .01, and no environment × time interaction, F(1,58) = .04, n.s. **OSpan**: ANOVA results showed a main effect of time, F(1,43) = 7.94, p < .01, & a time*env interaction, F(1,43) = 7.85, p < .01. Ospan improvement from pre-to-post-nature walk: t(22) = 4.08, p = .0005, d = 0.67.	n.s. †			n.s.					Ospan *; Change detection n.s
**Cimprich & Ronis** (2003)	RCT ⸙	185	Patients with newly diagnosed breast cancer, at a Univ. Medical center (100% female, mean age 53.8 years). USA.	Patient-led, home-based nature activities, vs logged relaxation time (control), 120 min/week, 36 day period.	**BDS, FDS, Necker cube pattern control (NCPC), Trail making test A (TMTA) & B (TMTB).**	**BDS:** Sign. better BDS-scores in nature intervention group than in the non-intervention group at T2 (p=.002), but intervention group also had sign better BDS scores at T1. . Differences in pre-post change are not reported. Similar pattern of results for FDS, TMTA & TMTB.	* / n.s.	* / n.s.			n.s.		* / n.s.	* / n.s.	
**Emfield**, Adam G.; Neider, Mark B. (2014)	RCT □	202	Univ students (128 female; mean age 19.8). Univ. Central Florida, USA.	7 conditions: natural or urban sounds, images of natural or urban environments, or a combination of both, or no exposure/control.	**BDS, ANT.** BDS: Adaptive version; DV= last string length in which two trials were correct, indicating the participant's digit span capacity.	Indications of practice effects were found, but no sign differential effects of environment condition on **BDS** or **ANT** measures.	n.s. †			n.s.					
**Gamble**, Howard Jr., & Howard (2014).	RCT □	26 + 30	Univ students at Georgetown University (mean age 20.54 years, SD 1.24).<br>30 older adults from the community (mean age 69.10 years, SD 3.92). Gender NR. USA.	6 min picture viewing- either 50 nature pictures or 50 urban pictures. Same pictures as Berman (2008) study.	**BDS**: 2-8 digit strings, 2 at each lenght; DV=nr of strings recalled correctly. **ANT.**	**BDS**: No difference in improvement between nature and urban conditions. Sign main effect of time (pre, post,) F(1, 52) = 25.80, p < .001. Pre-post contrasts for Nature: t(27) = −3.20, p = .004; Urban: t(27) = −3.99, p < .001. **ANT-E:** Sign time*env interaction, F(1, 52) = 6.88, p = 0.01. Executive Attention improvement from pre-to-post-nature pictures: t(27) = 5.27, p < .001, d = 0.76. No sign time*env interactions were seen for the alerting and orienting attention components.	n.s. †			*					ANT-A & O: n.s.

**Gidlow**, CJ., Jones, MV., Hurst, G., et al. (2016).	RCrT ⸙	38	Adults (35% female; mean age 40.9 years, SD 17.6). West Midlands, UK.	30-min walks: residential (urban), natural (green), and natural with water (blue). Pre, post, and delayed (30 min) post walk measure.	**BDS:** 3-9-digt strings. Stop after 2 failures. DV=length of the longest correct sequence.	**BDS**: Iinteraction effect of time*environment a significant environment*time , F(4, 148)=2.89, p= h2 ¼ 0.02, such that improvements in cognitive task performance persisted at T3 following exposure to both natural environments, but reduced to below baseline levels in the urban condition: Blue vs. Urban (T1 vs. T3), F(1, 37) = 9.26, p = .004; Green vs. Urban (T2 vs. T3), F(1, 37) = 4.35, p =.044.	* †								
**Li**, D., & Sullivan, W. C. (2016).	RCT □	94	High school students across 3 conditions (53 females, age NR). No window=32; , barren window=32; and greenwindow=30. Five public high schools in central Illinois, USA.		**BDS, FDS.**	Regression model on combined **FDS & BDS** score change: Students with green window views improved with (unstandardized B coefficient) 0.7 units more than their peers assigned to a barren window view (p<0.001), after controlling for the other variables. The difference in digit span score changes/restoration between the no window condition and the barren condition was not significantly different (p = 0.67). Mean BDS scores are not reported.			* †						
**Lin**, Y-H., Tsai, C-C; Sullivan, et al. (2014)	RCT □	138	138 univ undergraduate students (73 females, age NR). National Taiwan University, Taiwan.	Image conditions (5 images, total expo 100 sec): (a) streetscapes with absolutely no trees; (b) streetscapes with flashes of trees in which participants had minimal awareness of the content; (c) streetscapes with trees; and (d) streetscapes with trees to which participants were told to pay attention.	**BDS:** 4-10 digits (2 trials at each length); DV= length of longest correct sequence recited.	Awareness level had an effect on **BDS** score change (difference between BDS pre-test and post-test) even after controlling for participant's baseline (pre-test BDS), where F(3, 133) = 11.84, p < 0.001. Paired comparison showed that BDS change in the Heightened Awareness treatment was sig greater than in all other groups. There was no difference between the Moderate Awareness and the Minimal Awareness treatments, but both groups' scores were higher than the No Tree treatment group.	* †								
**Perkins et al.** (2011)	RCT ⸙	26	Univ. Students, (73 % female, age 19-24 years). USA.	Walk in natural (woods) vs urban (residential vs parking lot) setting, 20 min.	**BDS, FDS, Logical Memory.**	No sign difference between nature & urban exposures in **BDS** score changes, nor **FDS** or L**ogical memory.**	n.s.	n.s.							Logical memory n.s.
**Rogerson**, M., & Barton, J. (2015).	RCrT □	12	Adults from Univ. of Essex (50% female, mean age 27.8 years, SD 5.5). UK.	Visual exercise environments (**video**: forest run vs Boston marathon route/ control), during Exercise at 60% VO2peak for 15-mins.	**BDS:** 3-11 digits. DV=max string-length recited. Cognitively fatiguing tasks were done before pre-excercise BDS testings.	Time*Env interaction for **BDS** (F2,22 = 6.267, p = 0.007). Scores sign. improved in the nature condition (p < 0.001) but did not in the built or control conditions.	* †								
**Stark et al.** (2003)	cluster RCT ⸙	57	57 pregnant women from a prenatal class (mean age 29.1 years). USA.	Outdoor activitites vs discussion on pregnancy discomforts.	**BDS, FDS, Category Matching, TMTA, TMTB, Error Scale.**	No sign effect on **BDS** (Test statistics not reported). Sign better **Error scale** performance after nature but the time*env interaction is NR.	n.s.	n.s.					n.s.	n.s.	Error Scale */n.s; Category matching n.s.
**Triguero-Mas** M, Gidlow CJ, Martínez D, et al. (2017)	RCrT. ⸙	26	Adults (15 felames, median age 44.32). Barcelona, Spain.	Walks in Green, Blue, & Urban environments in groups of 2–6. BDS at time 1, 3, 5, 7, 9.	**BDS**: 3-9 digit strings (two of each length). Test stop after 2 consecutively errors. DV= total nr of correct sequences recalled.	**BDS**: Multilevel mixed-effects linear regression models were used with subject and BDS baseline levels at time 1 entered as random effects to evaluate the impact of exposure environment on changes in BDS: Coefficients (CI)and p-values (urban env exp is the reference): Green: -0.38 (-0.97, 0.21), p=0.20. Blue: 0.19 (-0.39, 0.77), p=0.52. Mean BDS scores are not reported.	n.s.								

Table 12 continued.

Studies using only other executive cognitive tests							BDS	FDS	FDS & BDS	ANT-E	NCPC	SART	TMTA	TMTB	Other
**Berman et al.** (2008, exp 2)	RCrT □	12	Univ students, (75% female, mean age 24.25 years). University of Michigan, USA.	10-min nature vs urban picture viewing.	BDS, **ANT (executive, alerting, orienting components).**	**ANT:** The picture type-by-time interaction was of most interest and was significant only for the executive portions of the ANT according to predictions, whereby pictures of nature led to more improved executive attention performance than did exposure to urban pictures, $F(1, 10) = 17.089$, prep =0.99.	(*)			*					ANT alerting & orienting: n.s.
**Berto** (2005, exp 1)	RCT □	32	Students (50% female, mean age 23 years). Italy.	Viewing images, natural<br>Viewing images, urban<br>25 images, displayed 15 sec each.	**Sustained Attention to Response Task (SART):** DVs reaction time, d-prime, correct & incorrect responses.	**SART:** Reported sign lower reaction times at T2 after the nature conditions, compared to the urban: $t(30) = -2.19$; $p = .03$. No group differences at T2 on Nr of correct or incorrect responses, or d-prime. Time*env interaction NR.						*/ n.s.			
**Berto** (2005, exp 3)	RCT □	32	Students (50% female, mean age 22 years). Italy.	Viewing images, natural<br>Viewing images, urban<br>25 images for duration of their choice.	**SART**	**SART:** Reported no sign difference between env groups on reaction time, the Nr of correct or incorrect responses or d-prime.						n.s.			
**Chen,** Lai & Wu (2011, exp 1)	RCT □	48	Senior secondary school students (58% female, age NR/range about 16-19 years). China.	Viewing images: natural, city, urban nightscape, sports.<br>For each condition: 10 images x 15 sec each.	**Colored number pictures (DV=reaction time)**	Env (nature, city, urban nightscape, sports)*Time (T1, T2, T3): $F = 8.27$; $p < .001$. Reaction times increased in the city cond., and decreased in the nature cond. and in the urban night cond. (greatest RT decrease) from T1-T3.									Colored nr pic * (mixed findings)
**Evensen,** K.H., Raanaas, R.K. Et al. (2015)	RCT ⸙	85	85 univ. students (out of which 34 from Raanas et al. 2011): 57 F, 28 M, Age M=24.9 y, SD=5.7 y. Norway.	Office setting during cognitive tasks: pants vs inanimate objects * window vs no window. Measures at T1/baseline, T2/after a demanding task, & T3/after a 5-min break.	**Reading Span Task (RST).**	No superior **RST** improvement in **plant condition vs inanimate objects condition** for neither window nor no-window condition.									RST n.s.
**Geniole**, SN.; David, J.P. F.; Euzébio, R.F.R. et al. (2016)	RCrT ⸙	31	Univ students (100% male, mean age 24.61, *SD* = 3.88). Ontario, Canada.	15 min walk. Nature: naturalized landfill area (visible methane gas pipes throughout). Urban: neighbouring urban area/city center.	**Stroop** (computerized, 3 keypress responses for 3 different font colors). DV= difference in RT between congruent vs incongruent trials.	An ANOVA with two within-subject factors (Location: urban vs. naturalized landfill; Time: prewalk vs. postwalk) on the **Stroop** scores revealed only a marginal effect of Time ($F_{1,30} = 3.15$, $p = 0.09$, $\eta p2 = .10$), with better attentional control after the walks than before the walks (other ps > .64)									Stroop n.s.
**Greenwood**, A., & Gatersleben, B. (2016)	RCT ⸙	120	College students (55% female, aged 16–18 years). South-West London, UK.	20 min outdoors in nature vs indoors, alone vs. with a friend vs. playing a game on a mobile phone. There were 20 participants in each experimental condition.	**NCPC**	Sign. main effect for time, with reduced **NCPC** scores/reversals in all conditions (Mpre = 5.54, SD = 2.16, Mpost = 4.2, SD = 2.14; $F(1,114) = 58.21$, $p < 0.001$, $\eta p^2 = 0.34$). Sign time*env interaction, with NCPC scores reducing more in the outdoor env (Mpre = 5.76, SD = 1.99, Mpost = 3.84, SD = 1.69) than in the indoor env (Mpre = 5.32, SD = 2.32, Mpost = 4.59, SD = 2.46; $F(1,114) = 11.85$, $p < 0.001$), $\eta p^2 = 0.09$. There was a marginally significant interaction effect for context ($F(2,114) = 2.71$, $p = 0.07$, $\eta p^2 = 0.05$). Whilst mean reductions in scores were greater in the 'with a friend' context (M = −1.70, SD 1.89) and the 'alone' context (M = −1.50, SD 1.71) than the 'with a phone' context (M = −0.76, SD = 2.27), they were only significantly so in the 'with a friend' context compared with 'with a phone' ($t(78) = 2.01$, $p = 0.05$). There was					*				

						no sign 3-way interaction effect between env and context over time (F(2,114) = 2.00, p = 0.78).									
**Hartig et al.** (1991, exp 2)	RCT ⸙	102	102 students (50% female, mean age 20 years).Univ. & local area, USA.	Walking in natural (regional park) vs urban (city centre) env, vs reading magazines in comfortable laboratory setting, for 40 min.	**Proofreading Task (% errors detected)**	Greater error detection in the nature compared to the urban and indoor conditions at T2 were reported: t(94) = 2.45; p < 0.01. But it is unclear which of the 3 env groups are contrasted in the t-test and baseline measurements were not reported.									Proofreading task */n.s.
**Hartig,** Evans, Jamner et al**.** (2003)	RCT ⸙	112	Students, Univ. & local area, USA. 50% female, mean age 20.8 years.	Sitting, natural view; then walking in nature reserve, vs. sitting, no view; then walking, urban (city streets), 1 h (10 min passive; 50 min active). Half of the participants performed fatiguing cognitive tasks for about 1 h before the view + walk.	**NCPC, Search and Memory Test.** Testing was done pre environment exposure (T1), mid walk (T2) & post-walk (T3).	**NCPC:** There were time*env interactions in favour of the nature group (incl. both the fatiguing task group and no task group) from T1-T2: F(1 ,98) = 13.15; p < 0.001; and from T1-T3: F(1,100) = 5.59; p = 0.02. **Search & Memory Test:** No sign effect of env or time*env interaction (results NR).					*				Search & memory test NR
**Jaggard,** Charles (2016), Master thesis, Indiana State University.	RCT □	109	University students, (81.7% female, mean age 19.2 years, range 18-43). Indiana, USA.	Viewed nature or urban pictures (5 pics for 1 min each= 5 min expo), with the instruction to watch the pics freely, vs. Instruction to direct their attention to the pictures.	**NCPC**	Free-Nature participants showed greatest recovery from directed attention fatigue (M = .55, SD = 1.95), followed by Free-Urban participants (M = .07, SD = .64), Directed-Urban participants (M = -.17, SD = 1.50), and finally with Directed-Nature participants exhibiting the least recovery of any group (M = -.41, SD = 1.41). Comparing pre-post scores, those watching freely showed greater recovery than those instructed to direct attention, t(86) = 2.21, p = .03. No significant differences were found between the "free" and "directed" urban conditions, t(86) = .58, p = .57, or between the Free-Nature and Free-Urban conditions, t(86) = 1.14, p =0.26.					n.s.				
**Johansson et al.** (2011)	RCrT ⸙	20	Univ students (50% female, mean age 24.2 years (males) & 22.4 years (females)). Sweden.	Walking, natural (park) vs. Urban (streets) for 40 min. Four walks, 1 week apart (natural vs urban, alone vs with a frieand).	**Symbol Substitution Test.**	A sign time*env interaction was found, whereby the nr of correct substitutions decreased more after the nature walk than the urban (combined conditions of being alone and with friend): F(1, 18) = 5.99, p = .025, Eta^2 = 0.250. Higher baseline/T1 scores in the nature condition suggest regression to the mean may explain results, according to the authors.									Symbol substitution: (reverse effect)
**Laumann et al.** (2003)	RCT □	28	Univ students (100% female, age 18-24 years). Norway.	Watching video, natural (island waterside) Watching video, urban (city streets) 80 scenes x 15 sec each.	**ANT-** Orienting component.	No raw data or statistical tests for type of stimuli by time effects on cognitive performance are presented.									ANT-orienting: NR
**Lee**, K. E., Williams, K. J., Sargent, L. D., et al. (2015)	RCT □	150	150 Univ.students (71% female, mean age 20). Melbourne, Australia.	40 sec viewing of flowering meadow green roof or a bare concrete roof.	**SART:** DVs included SD in response times, fast frequency variability in response times, slow-frequency/gradual changes in response variabilty,commision & omission errors.	Green roof viewing resulted in lower omission errors, and lower response variability, but not concrete roof viewing. **SD in response times:** Reduced response variability was seen post green roof viewing [F(1, 141) = 5.00, p =0.027, r =0.19], while response variability increased pre to post viewing of the concrete roof [F(1, 141)=7.86, p=0.006, r=0..23]. **Fast-frequency response variability** in 2nd half of the test, post treatment, was higher in the concrete roof group compared to the green roof group [U=1994, p=0.012, r=0.19.] **Response time:** no overall difference in mean response times for the green or concrete roof groups [F (1, 145)=0.10, p=0 .754], but results are not distinguished for pre vs post exposure testings. **Omission errors**: Post exposure, in the 2nd half of the						*/ n.s.			

						task, the concrete roof group made more omission errors than the green roof group [U=2318, p=0 .041, r=0.14]. No group differences or treatment effects were seen for **commission errors**. It is reported that the treatment groups did not differ in the DVs at baseline, but time*env interactions are not reported for any dependent variable.									
**Raanaas**, R. K., Evensen, K. H., Rich, D., et al. (2011)	RCT ⸙	34	34 Univ students (22 females, mean age= 25.0 , SD = 5.8; 12 males, mean age=23.3, SD = 3.9). Norway.	Office setting with four indoor plants vs. without plants (all with windows). Measures at T1, T2 (after proofreading task), T3 (after 5 min rest).	**RST** (12 four-sentence trials, then 8 six-sentence trials).	**RST** performance improved from T1-T2 in plant condition, but not in the no-plant condition. Neither group improved performance from T2-T3. Time* env interaction effect NR.									RST */ n.s.
**Sahlin,** E., Lindegård, A., Hadzibajramovic, E., et al. (2016)	RCrT ⸙	51	51 adults (39 females, mean age 45 years, age range 21-72). Sweden.	Relaxation excercise outdoors in nature, vs. Indoors.	**NCPC**	A sign. main effect of env condition was seen on NCPC in favour of the outdoor condition (p = 0.012), but the time*env interaction effect was not significant.					*/ n.s.				
**Sonntag-Öström**, E., Nordin, M., Lundell, Y., et al. (2014)	RCrT ⸙	20	20 exhaustion patients (100% females, mean age 41.6, SD 7.3), Sweden.	3 forest & one city environment in randomized order. Approx. 50 min duration in each env.	**NCPC.** DV: unfocused & focused reversals.	Reported less **NCPC** reversals in forests vs city env, but the Time*Env interaction is not reported.					*/n.s.				
**Shin et al.** (2011)	RCrT ⸙	60	Univ students (58% male, mean age 23.3 years). South Korea.	Natural (park) vs urban (city streets) walks, 50-55 min. Two of each walk.	**TMTB (DV=completion time)**	**TMTB** scores/completion time at T1 vs T2 for each env and walk indicate faster completion time after the nature walks but not after the urban walks, but no test statistics are reported.								NR	

[a] Excluding studies/results on BDS by Berman et al. covered in Tables 1-3.

† = Descriptive statistics for BDS were reported in the original research paper and are presented in Table 17.

⸙ = Real environments are used in the environment exposure conditions.

□ = Artificial environments are used in the environment exposure conditions, including virtual reality, pictures or videos of real environments.

* = a statistically significant effect of environment condition, in favour of nature exposure, on the cognitive outcome measure is reported at alpha ≤ 0.05. Note that not all such reported effects are in the form of an environment by time interaction. */n.s.= a significant effect of the nature environment on cognitive performance is reported but there is no significant time*environment interaction or this is not reported at all; M=mean. SD=standard deviation. NR=not reported in the original research report.

Table 13. Descriptive statistics for mean Backwards Digit Span (BDS) scores by time and environment condition, for studies by Berman et al. and all other studies identified when reviewing the literature.

Table 13. Descriptive statistics for mean Backwards Digit Span (BDS) scores by time and environment condition, for studies by Berman et al. and all other studies identified when reviewing the literature.

			NATURE						URBAN						OTHER/CONTROL						
***Studies by Berman et al.* ǂ**			**Pre Nature**		**Post Nature**		**Change**		**Pre Urban**		**Post Urban**		**Change**		**Pre Control**		**Post Control**		**Change**		**Reported results/ comment**
**Author, year (experiment), type of environment exposure, location**	**Design (BDS measure)**	**N**	**M**	**SD**	**M**	**SD**	**M**	**SD**	**M**	**SD**	**M**	**SD**	**M**	**SD**	**M**	**SD**	**M**	**SD**	**M**	**SD**	
**Berman et al. (2008(1))**. Walk, UM.	RCrT (ǂ)	37	7.86	2.28	9.43	2.48	1.57	1.99	7.86	1.80	8.43	2.02	0.57	2.02	.	.	.	.	.	.	The location-by-time interaction was sign., F(1, 36)= 6.055, p_{rep} = 0.95, indicating that improvement on BDS was greater after the nature walk than the urban walk.
**Berman et al. (2008(2))**. Picture study, UM.	RCrT (ǂ)	12	7.92	3.32	9.33	2.96	1.42	1.68	7.83	3.59	8.83	3.13	1.00	2.41	.	.	.	.	.	.	The location-by-time interaction was not sign., F(1, 10) = 0.486, p_{rep} = 0.68. But there was sign. Improvement in BDS after the nature (t(11) = 2.972, p_{rep} = 0.96) but not the urban (t(11)=1.436, p_{rep}=0.83) image condition.
**Berman et al. 2011**. Walk, healthy sample, UM.	RCrT (ǂ)	21	8.27	3.08	9.54	2.66	1.26	1.89	8.46	2.63	9.82	2.39	1.36	2.46	.	.	.	.	.	.	New / unpublished data.
**Berman et al. (2012)**. Walk, MDD sample, UM.	RCrT (ǂ)	19	7.42	3.01	8.63	2.87	1.21	1.44	8.20	2.48	7.84	2.24	-0.36	1.03	.	.	.	.	.	.	A time-by-location interaction was found, F(1, 18)=20.5, p<.001, η_p^2=0.53, indicating that improvement on BDS was greater after the nature than the urban walk. BDS performance improved sign. after the nature walk, t(18)=3.67, p<0.005, while there was a trending decrease in BDS performance after the urban walk, t(18)=−1.91, p=0.07.
**Berman et al. 2015.** Picture study, UC.	RCrT (ǂ)	45	9.24	2.19	9.47	2.14	0.22	2.25	9.02	2.39	9.02	2.29	0.00	1.99	.	.	.	.	.	.	New / unpublished data.
**Berman et al. 2015.** Picture study, UM.	RCrT (ǂ)	37	8.62	2.55	9.30	2.34	0.68	1.80	9.00	2.36	9.19	2.36	0.19	2.09	.	.	.	.	.	.	New / unpublished data.
**Berman et al. 2016.** Walk, UC.	RCrT (ǂ)	49	9.65	3.15	10.18	3.10	0.53	2.48	9.39	2.60	9.49	2.91	0.10	2.03	.	.	.	.	.	.	New / unpublished data.
**Berman et al. 2016.** Virtual Reality study, UC.	RCrT (ǂ)	82	9.71	2.77	10.34	2.83	0.63	2.11	9.67	2.61	10.28	2.66	0.61	2.16	.	.	.	.	.	.	New / unpublished data.
**Berman et al. 2016.** Virtual Reality study- with habituation, UC.	RCT (ǂ)	N=42; U=40; C(habituation)=82	9.95	2.41	10.12	2.84	0.17	2.09	10.40	2.85	9.85	2.76	-0.55	2.10	8.87	2.44	9.66	2.37	0.79	2.26	New / unpublished data.
**Van Hedger et al. (2018)**. Composite study- Sounds. UC.	RCT (ǂ)	Nat=22; U=22	9.77	2.56	10.64	2.52	0.86	2.14	8.50	3.19	8.77	2.88	0.27	2.55	.	.	.	.	.	.	Published in: Van Hedger, Nusbaum, Clohisy, et al. (2018)
**Van Hedger et al. 2016.** Composite study- Pictures. UC.	RCT (ǂ)	Nat=19; U=21	9.42	2.67	9.95	2.20	0.53	2.25	8.33	2.58	9.19	2.64	0.86	2.20	.	.	.	.	.	.	New / unpublished data.
**Bourrier et al. (2018).** Video study, UBC.	RCT (ǂ)	Nat=30; U=30; C=30	7.53	2.78	8.83	2.52	1.30	2.05	7.73	3.17	7.80	3.31	0.07	2.85	7.17	3.17	7.70	2.79	0.53	2.39	Published in: Bourrier, Berman & Enns (2018)

Grand means, sample size weighted, for studies above (incl. in pooled-analyses):	RCT's & RCrT's	Nat=415; Urb=415; Control=112	9.02	2.80	9.77	2.68	0.75	2.11	8.97	2.72	9.26	2.71	0.29	2.19	8.41	2.75	9.13	2.63	0.72	2.29	Note: Grand means for nature & urban conditions are based on observations from both 1st & 2nd sessions. Grand means for "other control" conditions are based on 2 studies where all observations are from 1st sessions.
			**NATURE**						**URBAN**						**OTHER/CONTROL**						
Studies of BDS identified in the literature review			**Pre Nature**		**Post Nature**		**Change**		**Pre Urban**		**Post Urban**		**Change**		**Pre Control**		**Post Control**		**Change**		**Reported results/ comment**
**Author, year, type of environment exposure/ subgroup.**	**Design (BDS measure)**	**N**	**M**	**SD**	**M**	**SD**	**M**	**SD**	**M**	**SD**	**M**	**SD**	**M**	**SD**	**M**	**SD**	**M**	**SD**	**M**	**SD**	
**Bodin, 2003**. Park vs city run. Men	RCrT (ǂ)	12 (6 men)	10.83	2.32	11.92	2.31	1.09	NR	11.92	2.76	12.17	2.89	0.25	NR	.	.	.	.	.	.	The sum of BDS & Forward digit span (FDS) was reported (max score 28). Differemce in change between nature and urban exposures (men & women): F(1, 10)=0.92; p=0.36.
Ibid. Women		(6 women)	11.67	3.82	10.58	3.68	-1.09	NR	10.67	2.89	11.25	3.22	0.58	NR	.	.	.	.	.	.	
**Bratman et al. 2015**. Walk.	RCT (ǂ)	60 (Nat=30; Urb=30)	6.80	2.24	7.50	2.46	0.70	NR	6.93	2.03	7.73	2.15	0.80	NR	.	.	.	.	.	.	Time*Env ANOVA: Time, F=8.4, p<0.01; Env, =0.72, ns; time*env, F=0.04, ns.
**Cimprich, 2003**. Nature activities vs relaxation time.	RCT (NR)	185 (Nat=83; Control=74)	4.99	0.15	5.20	0.14	0.21	NR	.	.	.	.	.	NR	4.51	0.13	4.58	0.14	0.07	NR	Std.errors are reported, not SD. Sign. diff. between environment groups in post-scores (p=.002). Differences in pre-post change not reported.
**Emfield et al. 2014**. Sounds.	RCT (¥)	Sounds (Nat=28; Urb=28; Control=27)	5.30	1.44	6.11	1.42	0.81	NR	5.75	1.04	5.79	1.23	0.04	NR	4.96	1.14	5.24	1.09	0.28	NR	Indications of practice effects were found, but no sign differential effects of environment/stimuli condition on cognitive task performance.
Ibid. Pictures	RCT (¥)	Images (Nat=27; Urb=27)	5.28	0.98	5.52	1.33	0.24	NR	5.64	1.22	5.60	1.23	-0.04	NR	.	.	.	.	.	.	
Ibid. . Audiovisual.	RCT (¥)	Images + sounds (Nat=28; Urb=27)	5.40	1.16	5.88	1.20	0.48	NR	5.52	1.16	5.44	1.25	-0.08	NR	.	.	.	.	.	.	
**Gamble et al.** 2014. Picture viewing. †	RCT (ǂ)	26 young, 30 old. (Nat=28; Urb=28)	NR	NR	NR	NR	(0,87)	NR	NR	NR	NR	NR	(0,87)	NR	.	.	.	.	.	.	No sign. time*env interaction on BDS. Pre-post contrasts: Nature: t(27) = −3.20, p = 0.004; Urban: t(27) = −3.99, p < .001. Main effect of time (pre, post,) F(1, 52) = 25.80, p < .001. Overall, older adults (M = 6.90, SD = 2.58) had lower BDS than young (M = 8.64, SD = 2.58), and BDS was higher post-picture viewing (M = 8.14, SD = 2.72) than pre-picture viewing (M = 7.27, SD = 2.66). The interaction of Age × Session was not sign., p = .72, and no sign. effects of env. type. All 4 groups (young vs old * nature vs urban), showed practice effects. †Mean BDS scores pre- & post each env condition are not reported, only pre & post scores across environment conditions.
**Gidlow et al. 2016**. Green Nature. T1-T2	RCrT (¥)	38. Green nature (T1, T2, T1-T2)	6.21	2.93	6.37	2.57	0.16	NR	6.68	5.79	6.84	2.52	0.16	NR	.	.	.	.	.	.	Blue vs. Urban (T1 vs. T3), F(1, 37) = 9.26, p = .004; Green vs. Urban (T2 vs. T3), F(1, 37) = 4.35, p =0.044. No other test statistics are reported for BDS.
Ibid. Green Nature. T3.		Green nature (T3, T1-3)			6.82	6.82	0.61	NR	.	.	6.45	2.35	-0.23	NR	.	.	.	.	.	.	
Ibid. Blue Nature. T1-T2		Blue Nature (T1, T2, T1-T2)	5.82	2.68	6.53	2.48	0.71	NR	.	.	.	.	.	.	.	.	.	.	.	.	
Ibid. Blue Nature. T3		Blue Nature (T3, T1-T3)			6.71	2.54	0.89	NR	.	.	.	.	.	.	.	.	.	.	.	.	

**Li, D., & Sullivan, W. C., 2016**. Window views. †	RCT (NR)	94. No window=32; , barren window=32; and green window=30.	NR	NR	NR	NR	(0,7 units)	NR	NR	NR	NR	NR	NR	NR	NR	NR	NR	NR	NR	NR	Regression model with DV digit span score (forwards & backwards) change: Students with green window views improved with (unstandardized B coefficient) **0.7 units** more than their peers assigned to a barren window view (p<0.001), after controlling for the other variables. †NB: Mean digit span scores/change are not reported, only digit span unit change in nature compared to urban condition.
**Lin et al. 2014**.	RCT (¥) 3 awareness levels.	Minimal awareness, Nat=31; Urb=34.	6.45	NR	7.06	NR	0.61	NR	7.12	NR	6.53	NR	-0.59	NR	.	.	.	.	.	.	Pre-score compared to post-score (negative=improvement). Low awareness: No nature: T= 2.385*; Nature: T= -2.31*.
Ibid.		Medium awareness, Nat=36	6.28	NR	6.83	NR	0.55	NR	.	.	.	.	.	.	.	.	.	.	.	.	Medium awareness, Nature pre-post: T= -2.28*
Ibid.		High awareness, Nat=37	6.57	NR	8.05	NR	1.48	NR	.	.	.	.	.	.	.	.	.	.	.	.	High awareness, Nature pre-post: T= -5.68***
**Perkins, 2011**. Walk.	RCT (NR)	26	NR	NR	NR	NR	NR	NR	NR	NR	NR	NR	NR	NR	.	.	.	.	.	.	No sign difference between nature & urban exposures in BDS score changes. Test statistics not reported.
**Rogerson et al. 2015**. Video †	RCrT (¥)	12 (Nat + Urb + Control=12)	3.70	.	5.10	.	1.40	NR	4.00	.	4.20	.	0.20	NR	4.15	.	4.30	.	0.15	NR	Time*Env interaction for BDS (F2,22 = 6.267, p = 0.007). Scores sign. improved in the nature condition (p < 0.001) but did not in the built or control conditions. †Means were interpreted from the graph of means in the original research paper.
**Stark, 2003**. Outdoor activities vs indoor.	cluster RCT (NR)	57. Nat=29; Control=28.	5.10	1.30	5.40	1.60	0.30	NR	.	.	.	.	.	.	4.70	1.10	5.00	1.60	0.30	NR	No sign effect of nature compared to control on BDS changes. Test statistics NR. Means reported by Ohly et al. 2016.

† = Descriptive statistics are not reported in means pre- and post each environment condition. See comment in "Reported results".
ǂ = total nr of correct trials in the digit span test is the dependent variable.
¥ = maximal string length in the digit span test is the dependent variable.
M=mean; SD=standard deviation; NR=not reported; RCT=Randomized controlled trial; RCrT=Randomized Crossover Trial.
UC=University of Chicago; UM=University of Michigan; UBC=University of British Columbia.
* p ≤ 0.05, ** p ≤ 0.01, ***p≤0.001.

References in Table 12 & Table 13:

Berman, M. G., Jonides, J., & Kaplan, S. (2008). The cognitive benefits of interacting with nature. Psychol Sci, 19(12), 1207-1212.

Berman, M. G., Kross, E., Krpan, K. M., Askren, M. K., Burson, A., Deldin, P. J., . . . Jonides, J. (2012). Interacting with nature improves cognition and affect for individuals with depression. J Affect Disord, 140(3), 300-305.

Berto, R. (2005, Exp 1 & 3 (exp 2 not random allocation)). Exposure to restorative environments helps restore attentional capacity. Journal of Environmental Psychology, 25, 249-259.

Bratman, Daily, Levy, & Gross (2015). The benefits of nature experience: Improved affect and cognition. Landscape and Urban Planning, 138, 41-50.

Bodin, M., & Hartig, T. (2003). Does the outdoor environment matter for psychological restoration gained through running? Psychology of Sport and Exercise, 4, 141-153.

Bourrier, S.C., Berman, M.G., & Enns, J.T. (2018). Cognitive strategies and natural environments interact in influencing executive function. Frontiers in Psychology.

Chen, C., Lai, Y.-H., & Wu, J.-P. (2011, Exp 1). Restorative affections about directed attention recovery and reflection in different environments. Chinese Mental Health Journal, 25, 681-685.

Cimprich, B., & Ronis, D. L. (2003). An environmental intervention to restore attention in women with newly diagnosed breast cancer. Cancer Nursing, 26, 284-292.

Emfield, Adam G.; Neider, Mark B. (2014). Evaluating visual and auditory contributions to the cognitive restoration effect. Frontiers in Psychology, Vol 5.

Evensen, K. H., Raanaas, R. K., Hagerhall, C. M., Johansson, M., & Patil, G. G. (2015). Restorative elements at the computer workstation: A comparison of live plants and inanimate objects with and without window view. Environment and Behavior, 47(3), 288-303.

Gamble, K. R., Howard, J. H., & Howard, D. V. (2014). Not just scenery: Viewing nature pictures improves executive attention in older adults. Experimental Aging Research, 40, 513-530.

Geniole, Shawn N.; David, Joacy P. F.; Euzébio, Roseanne F. R.; Toledo, Beatriz Z. S.; Neves, Andrea I. M.; McCormick, Cheryl M. (2016) Restoring land and mind: The benefits of an outdoor walk on mood are enhanced in a naturalized landfill area relative to its neighboring urban area. Academic Journal Ecopsychology, Vol 8(2).

Gidlow, C. J., Jones, M. V., Hurst, G., Masterson, D., Clark-Carter, D., Tarvainen, M. P., ... & Nieuwenhuijsen, M. (2016). Where to put your best foot forward: Psycho-physiological responses to walking in natural and urban environments. Journal of Environmental Psychology, 45, 22-29.

Greenwood, A., & Gatersleben, B. (2016). Let's go outside! Environmental restoration amongst adolescents and the impact of friends and phones. Journal of Environmental Psychology, 48, 131-139.

Hartig, T., M. Mang, and G. W. Evans. (1991, Exp 2 (Exp 1 not random allocation)). Restorative effects of natural environment experiences. Environment and Behavior 23:3–26.

Hartig, T., Evans, G. W., Jamner, L. D., Davis, D. S., & Gärling, T. (2003). Tracking restoration in natural and urban field settings. Journal of Environmental Psychology, 23, 109-123.

Jaggard, Charles (2016). Master thesis: The Effect of Intentionally Engaging Attention when Viewing Restorative Environments: Exploring Attention Restoration Theory. Indiana State University.

Johansson, M., Hartig, T., & Staats, H. (2011). Psychological benefits of walking: Moderation by company and outdoor environment. Applied Psychology: Health and Well-Being, 3, 261-280.

Laumann, K., Gärling, T., & Stormark, K. M. (2003). Selective attention and heart rate responses to natural and urban environments. Journal of Environmental Psychology, 23, 125-134.

Lee, K. E., Williams, K. J., Sargent, L. D., Williams, N. S., & Johnson, K. A. (2015). 40-second green roof views sustain attention: The role of micro-breaks in attention restoration. Journal of Environmental Psychology, 42, 182-189.

Li, D., & Sullivan, W. C. (2016). Impact of views to school landscapes on recovery from stress and mental fatigue. Landscape and Urban Planning, 148, 149-158.

Lin, Ying-Hsuan; Tsai, Chih-Chang; Sullivan, William C.; Chang, Po-Ju; Chang, Chun-Yen (2014) Does awareness effect the restorative function and perception of street trees? Frontiers in Psychology, Vol 5.

Perkins, S., Searight, H., & Ratwik, S. (2011). Walking in a natural winter setting to relieve attention fatigue: A pilot study. Psychology, 2, 777-780.

Raanaas, R. K., Evensen, K. H., Rich, D., Sjøstrøm, G., & Patil, G. (2011). Benefits of indoor plants on attention capacity in an office setting. Journal of Environmental Psychology, 31(1), 99-105.

Rogerson, M., & Barton, J. (2015). Effects of the visual exercise environments on cognitive directed attention, energy expenditure and perceived exertion. International journal of environmental research and public health, 12(7), 7321-7336.

Sahlin, E., Lindegård, A., Hadzibajramovic, E., Grahn, P., Vega Matuszczyk, J., & Ahlborg Jr, G. (2016). The influence of the environment on directed attention, blood pressure and heart rate—An experimental study using a relaxation intervention. Landscape research, 41(1), 7-25.

Shin, W. S., Shin, C. S., Yeoun, P. S., & Kim, J. J. (2011). The influence of interaction with forest on cognitive function. Scandinavian Journal of Forest Research, 26, 595-598.

Sonntag-Öström, E., Nordin, M., Lundell, Y., Dolling, A., Wiklund, U., Karlsson, M., et al. (2014) Restorative effects of visits to urban and forest environments in patients with exhaustion disorder. Urban Forestry & Urban Greening, 13, 344–354.

Stark, M. A. (2003). Restoring attention in pregnancy: The natural environment. Clinical Nursing Research, 12, 246-265.

Triguero-Mas, M., Gidlow, C. J., Martínez, D., de Bont, J., Carrasco-Turigas, G., Martínez-Íñiguez, T., ... & Jones, M. V. (2017). The effect of randomised exposure to different types of natural outdoor environments compared to exposure to an urban environment on people with indications of psychological distress in Catalonia. PloS one, 12(3), e0172200.

Van Hedger, S. C., Nusbaum, H., Clohisy, L., Jaeggi, S. M., Buschkuehl, M., & Berman, M. (2018, March 23). Of cricket chirps and car horns: The effect of nature sounds on cognitive performance. Psychonomic bulletin & review, 1-9. Preprint at https://doi.org/10.17605/OSF.IO/F5HCZ
