# Supplementary material for: Positive Effects of Nature on Cognitive Performance Across Multiple Experiments: Test Order but Not Affect Modulates the Cognitive Effects
Source: Front Psychol. 2019 Jul 3;10:1413. doi: 10.3389/fpsyg.2019.01413 (PMC6616085; doi:10.3389/fpsyg.2019.01413)
Supplement: Supplementary file 3 [file Table_3.pdf]

**Appendix C**

**Supplementary mediational path analyses:**

Due to the clear practice effects on BDS performance in the 1st test sessions, while the 2nd test sessions were devoid of such initial practice effects, the extent to which changes in affect may mediate the changes in BDS performance due to the respective environment exposures may only be adequately evaluated in the second test sessions. Furthermore, the results from the analyses of all study samples stratified by test session showed that the effect of environment condition on changes in BDS performance (i.e. the time × environment interaction) was significant only in the second test sessions ($p<0.001$), and a similar pattern was observed for positive affect ($p<0.01$). Hence, the primary conditions under which mediation may occur were only present in the second test sessions (i.e. there was an effect of the environment conditions on changes in the target outcome, BDS performance, and on changes in the postulated mediator, positive affect). Hence, for the purpose of testing if changes in positive affect mediates changes in BDS performance in the present study, the most relevant mediational path analyses are those of the changes in BDS and positive affect after each respective environment exposure tested in the *second test sessions*.

Since the effect of time × environment condition on negative affect was very small and not significant in neither the 1st ($p=0.300$) nor 2nd test sessions ($p=0.312$), it is not plausible that changes in BDS performance due to the environment conditions are mediated by changes in negative affect.

For clarity, mediational path analysis was therefore performed on the data from all the study samples, stratified by first versus second test sessions and environment condition, testing if and the extent to which changes in BDS were mediated by changes in positive affect.

Mediational path analyses for within-subjects/repeated measurements were performed using ordinary least squares regression (OLS) according to the procedure illustrated in Montoya and Hayes (2017), which implements the method described by Judd, Kenny, and McClelland (2001). The analyses were performed in SPSS 25 using the MEMORE-macro v.2.1 for SPSS (Montoya and Hayes, 2017).

**Results from the path analyses**

The results from the path analyses of primary interest are illustrated in the diagram below, which shows the direct, indirect (mediated by positive affect) and total effects of nature versus urban environment interactions (i.e. pre- to post effects) on BDS performance, testing if and the extent to which changes in BDS performance are mediated by changes in positive affect.

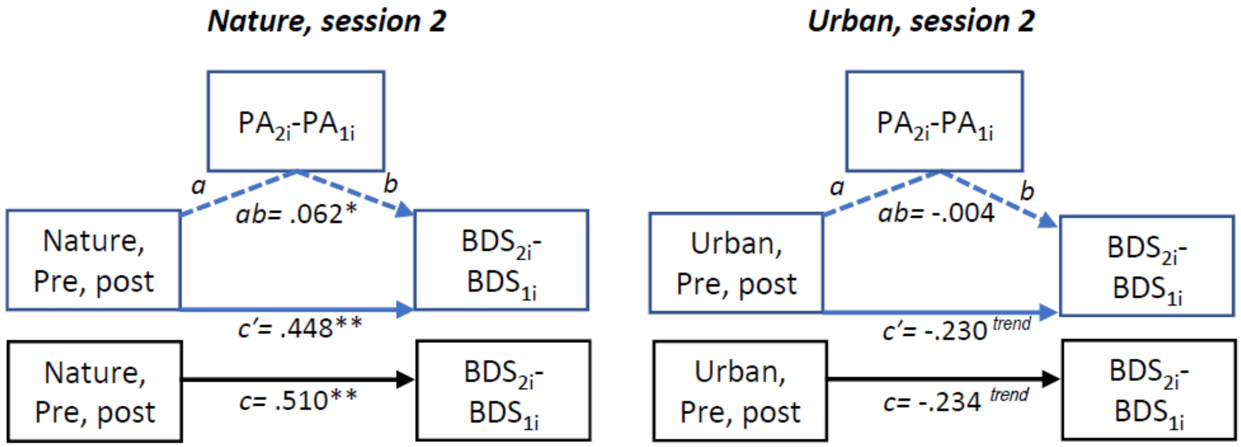


**Mediational path analyses of total, direct & indirect effects on BDS performance, via positive affect (PA), following nature vs urban environment interactions, in 2nd test sessions.**

Nature condition, 2nd test sessions:
Total effect, c: 0.510 *(SE: 0.154, t=*3.320*, df=194, 95% CI: 0.207, 0.813);*
Direct effect, c′: 0.448 *(SE: 0.155, t= 2.885, df=192, 95% CI: 0.142, 0.755);*
Indirect effect, ab: 0.062 *(SE: 0.038, 95% CI: 0.001, 0.145).*

Urban condition, 2nd test sessions:
Total effect, *c*: -0.234 *(SE: 0.137, t=* -1.714*, df=187, 95% CI: -0.504, 0.035);*
Direct effect, c′: -0.230 *(SE: 0.138, t= -1.670, df= 185, 95% CI: -0.503, 0.042)*;
Indirect effect, *ab*: -0.004 *(SE: 0.019, 95% CI: -0.050, 0.032).*
*** $p< 0.01$, * $p<0.05$, trend $p<0.10$*

In the following results section, the complete results from the path analyses are shown, and for reference includes both the analyses for nature versus urban conditions in 2nd test sessions (1-2), and in 1st test sessions (3-4).

The variables for BDS performance and positive affect in the analyses are the following:

BDS_t1= BDS performance at time 1, before an environment interaction

BDS_t2= BDS performance at time 2, after an environment interaction

PA_t1= positive affect score at time 1, before an environment interaction

PA_t2= positive affect score at time 2, after an environment interaction

**1. NATURE CONDITION, SESSION 2**
**-CHANGES IN BDS AND POSITIVE AFFECT FROM PRE- TO POST NATURE ENVIRONMENT EXPOSURES**

```
MODEL: EFFECT OF TIME ("X") ON THE OUTCOME BDS PERFORMANCE ("Y") AND
    THE MEDIATOR POSITIVE AFFECT ("M")


Run MATRIX procedure:

****************** MEMORE Procedure for SPSS Version 2.1 ******************

                           Written by Amanda Montoya

                    Documentation available at akmontoya.com

**************************************************************************
NATURE CONDITION, SESSION 2.

Model:
  1

Variables:
Y = BDS_t2   BDS_t1
M = PA_t2    PA_t1

Computed Variables:
Ydiff =         BDS_t2    -       BDS_t1
Mdiff =         PA_t2     -       PA_t1
Mavg =  (       PA_t2     +       PA_t1    )        /2       Centered

Sample Size:
  195

**************************************************************************
Outcome: Ydiff =  BDS_t2    -       BDS_t1

Model
       Effect         SE          t          p       LLCI       ULCI
'X'      ,5103      ,1537     3,3202      ,0011      ,2072      ,8134

Degrees of freedom for all regression coefficient estimates:
  194

**************************************************************************
Outcome: Mdiff =  PA_t2     -       PA_t1

Model
       Effect         SE          t          p       LLCI       ULCI
'X'      ,1297      ,0490     2,6484      ,0088      ,0331      ,2264

Degrees of freedom for all regression coefficient estimates:
  194

**************************************************************************
Outcome: Ydiff =  BDS_t2    -       BDS_t1
```

Model Summary

| R | R-sq | MSE | F | df1 | df2 | p |
|---|---|---|---|---|---|---|
| ,1562 | ,0244 | 4,5400 | 2,4001 | 2,0000 | 192,0000 | ,0934 |

Model

| | coeff | SE | t | p | LLCI | ULCI |
|---|---|---|---|---|---|---|
| 'X' | ,4481 | ,1553 | 2,8846 | ,0044 | ,1417 | ,7545 |
| Mdiff | ,4791 | ,2245 | 2,1344 | ,0341 | ,0364 | ,9219 |
| Mavg | -,1336 | ,1968 | -,6790 | ,4979 | -,5217 | ,2545 |

Degrees of freedom for all regression coefficient estimates:
192

****************** TOTAL, DIRECT, AND INDIRECT EFFECTS *******************

**Total effect of X on Y**

| Effect | SE | t | df | p | LLCI | ULCI |
|---|---|---|---|---|---|---|
| ,5103 | ,1537 | 3,3202 | 194,0000 | ,0011 | ,2072 | ,8134 |

**Direct effect of X on Y**

| Effect | SE | t | df | p | LLCI | ULCI |
|---|---|---|---|---|---|---|
| ,4481 | ,1553 | 2,8846 | 192,0000 | ,0044 | ,1417 | ,7545 |

**Indirect Effect of X on Y through M**

| | Effect | BootSE | BootLLCI | BootULCI |
|---|---|---|---|---|
| Ind1 | ,0622 | ,0380 | ,0009 | ,1452 |

Indirect Key
Ind1 'X' -> Mdiff -> Ydiff

********************* ANALYSIS NOTES AND WARNINGS *********************

Bootstrap confidence interval method used: Percentile bootstrap.

Number of bootstrap samples for bootstrap confidence intervals:
5000

The following variables were mean centered prior to analysis:
( PA_t2 + PA_t1 ) /2

Level of confidence for all confidence intervals in output:
95,00

------ END MATRIX -----

**2. URBAN CONDITION, SESSION 2**
**-CHANGES IN BDS AND POSITIVE AFFECT FROM PRE- TO POST URBAN ENVIRONMENT EXPOSURES**

MODEL: EFFECT OF TIME ("X") ON THE OUTCOME BDS PERFORMANCE ("Y") AND THE MEDIATOR POSITIVE AFFECT ("M")

************** MEMORE Procedure for SPSS Version 2.1 *******************

Written by Amanda Montoya

```
                    Documentation available at akmontoya.com

****************************************************************************
URBAN CONDITION, SESSION 2.

Model:
  1

Variables:
Y = BDS_t2   BDS_t1
M = PA_t2    PA_t1

Computed Variables:
Ydiff =         BDS_t2    -       BDS_t1
Mdiff =         PA_t2     -       PA_t1
Mavg =  (       PA_t2     +       PA_t1    )        /2       Centered

Sample Size:
  188

****************************************************************************
Outcome: Ydiff =  BDS_t2    -       BDS_t1

Model
        Effect         SE          t          p       LLCI       ULCI
'X'     -,2340      ,1366    -1,7137      ,0882     -,5035      ,0354

Degrees of freedom for all regression coefficient estimates:
  187

****************************************************************************
Outcome: Mdiff =  PA_t2     -       PA_t1

Model
        Effect         SE          t          p       LLCI       ULCI
'X'     -,0734      ,0477    -1,5379      ,1258     -,1676      ,0208

Degrees of freedom for all regression coefficient estimates:
  187

****************************************************************************
Outcome: Ydiff =  BDS_t2    -       BDS_t1

Model Summary
          R       R-sq        MSE          F        df1        df2          p
      ,0579      ,0033     3,5325      ,3107     2,0000   185,0000      ,7333

Model
           coeff         SE          t          p       LLCI       ULCI
'X'       -,2304      ,1379    -1,6700      ,0966     -,5025      ,0418
Mdiff      ,0501      ,2102      ,2383      ,8119     -,3645      ,4647
Mavg       ,1366      ,1840      ,7422      ,4589     -,2265      ,4997

Degrees of freedom for all regression coefficient estimates:
  185
```

```
******************** TOTAL, DIRECT, AND INDIRECT EFFECTS ******************
```

**Total effect of X on Y**

| Effect | SE | t | df | p | LLCI | ULCI |
|---|---|---|---|---|---|---|
| -,2340 | ,1366 | -1,7137 | 187,0000 | ,0882 | -,5035 | ,0354 |

**Direct effect of X on Y**

| Effect | SE | t | df | p | LLCI | ULCI |
|---|---|---|---|---|---|---|
| -,2304 | ,1379 | -1,6700 | 185,0000 | ,0966 | -,5025 | ,0418 |

**Indirect Effect of X on Y through M**

| | Effect | BootSE | BootLLCI | BootULCI |
|---|---|---|---|---|
| Ind1 | -,0037 | ,0191 | -,0503 | ,0317 |

```
Indirect Key
Ind1  'X'      ->       Mdiff    ->       Ydiff

********************** ANALYSIS NOTES AND WARNINGS *****************

Bootstrap confidence interval method used: Percentile bootstrap.

Number of bootstrap samples for bootstrap confidence intervals:
  5000

The following variables were mean centered prior to analysis:
 (        PA_t2     +       PA_t1    )        /2

Level of confidence for all confidence intervals in output:
      95,00

------ END MATRIX -----
```

**3. NATURE CONDITION SESSION 1,**
**-CHANGES IN BDS AND POSITIVE AFFECT FROM PRE- TO POST NATURE ENVIRONMENT EXPOSURES**

MODEL: EFFECT OF TIME ("X") ON THE OUTCOME BDS PERFORMANCE ("Y") AND THE MEDIATOR POSITIVE AFFECT ("M")

```
Run MATRIX procedure:

****************** MEMORE Procedure for SPSS Version 2.1 ******************

                           Written by Amanda Montoya

                    Documentation available at akmontoya.com

***************************************************************************
NATURE CONDITION, SESSION 1.

Model:
  1

Variables:
Y = BDS_t2   BDS_t1
```

```
M = PA_t2    PA_t1

Computed Variables:
Ydiff =         BDS_t2    -       BDS_t1
Mdiff =         PA_t2     -       PA_t1
Mavg =  (       PA_t2     +       PA_t1    )        /2       Centered

Sample Size:
  187

***************************************************************************
Outcome: Ydiff =  BDS_t2    -       BDS_t1

Model
        Effect         SE          t          p       LLCI       ULCI
'X'      ,9091      ,1516     5,9964      ,0000      ,6100     1,2082

Degrees of freedom for all regression coefficient estimates:
  186

***************************************************************************
Outcome: Mdiff =  PA_t2     -       PA_t1

Model
        Effect         SE          t          p       LLCI       ULCI
'X'     -,1107      ,0559    -1,9798      ,0492     -,2210     -,0004

Degrees of freedom for all regression coefficient estimates:
  186

***************************************************************************
Outcome: Ydiff =  BDS_t2    -       BDS_t1

Model Summary
          R       R-sq        MSE          F        df1        df2          p
      ,1472      ,0217     4,2508     2,0364     2,0000   184,0000      ,1334

Model
           coeff         SE          t          p       LLCI       ULCI
'X'        ,9315      ,1524     6,1133      ,0000      ,6309     1,2321
Mdiff      ,2024      ,1991     1,0166      ,3107     -,1904      ,5952
Mavg       ,3529      ,2190     1,6115      ,1088     -,0792      ,7850

Degrees of freedom for all regression coefficient estimates:
  184

****************** TOTAL, DIRECT, AND INDIRECT EFFECTS ********************
```

**Total effect of X on Y**

```
     Effect         SE          t         df          p       LLCI       ULCI
      ,9091      ,1516     5,9964   186,0000      ,0000      ,6100     1,2082
```

**Direct effect of X on Y**

```
     Effect         SE          t         df          p       LLCI       ULCI
      ,9315      ,1524     6,1133   184,0000      ,0000      ,6309     1,2321
```

**Indirect Effect of X on Y through M**

```
          Effect     BootSE   BootLLCI   BootULCI
Ind1      -,0224      ,0293     -,0921      ,0249

Indirect Key
Ind1  'X'      ->       Mdiff    ->       Ydiff

************************ ANALYSIS NOTES AND WARNINGS ************************
Bootstrap confidence interval method used: Percentile bootstrap.

Number of bootstrap samples for bootstrap confidence intervals:
  5000

The following variables were mean centered prior to analysis:
 (        PA_t2     +       PA_t1    )        /2

Level of confidence for all confidence intervals in output:
      95,00

------ END MATRIX -----
```

**4. URBAN CONDITION, SESSION 1**
**-CHANGES IN BDS AND POSITIVE AFFECT FROM PRE- TO POST URBAN ENVIRONMENT EXPOSURES**

```
MODEL: EFFECT OF TIME ("X") ON THE OUTCOME BDS PERFORMANCE ("Y") AND
    THE MEDIATOR POSITIVE AFFECT ("M")

Run MATRIX procedure:

******************* MEMORE Procedure for SPSS Version 2.1 ******************

                           Written by Amanda Montoya

                    Documentation available at akmontoya.com

*****************************************************************************
URBAN CONDITION, SESSION 1.

Model:
  1

Variables:
Y = BDS_t2   BDS_t1
M = PA_t2    PA_t1

Computed Variables:
Ydiff =         BDS_t2    -       BDS_t1
Mdiff =         PA_t2     -       PA_t1
Mavg =  (       PA_t2     +       PA_t1    )        /2       Centered

Sample Size:
  195

*****************************************************************************
Outcome: Ydiff =  BDS_t2    -       BDS_t1
```

```
Model
       Effect         SE          t          p       LLCI       ULCI
'X'     ,8141      ,1613     5,0463      ,0000      ,4959     1,1323

Degrees of freedom for all regression coefficient estimates:
  194

****************************************************************************
Outcome: Mdiff =  PA_t2     -       PA_t1

Model
       Effect         SE          t          p       LLCI       ULCI
'X'    -,1733      ,0541    -3,2011      ,0016     -,2801     -,0665

Degrees of freedom for all regression coefficient estimates:
  194

****************************************************************************
Outcome: Ydiff =  BDS_t2    -       BDS_t1

Model Summary
          R       R-sq        MSE          F        df1        df2          p
      ,0748      ,0056     5,0993      ,5396     2,0000   192,0000      ,5839

Model
          coeff         SE          t          p       LLCI       ULCI
'X'       ,8513      ,1659     5,1303      ,0000      ,5240     1,1787
Mdiff     ,2149      ,2149     1,0000      ,3186     -,2089      ,6387
Mavg      ,0474      ,2193      ,2161      ,8291     -,3851      ,4799

Degrees of freedom for all regression coefficient estimates:
  192

****************** TOTAL, DIRECT, AND INDIRECT EFFECTS ******************
```

**Total effect of X on Y**

```
     Effect         SE          t         df          p       LLCI       ULCI
      ,8141      ,1613     5,0463   194,0000      ,0000      ,4959     1,1323
```

**Direct effect of X on Y**

```
     Effect         SE          t         df          p       LLCI       ULCI
      ,8513      ,1659     5,1303   192,0000      ,0000      ,5240     1,1787
```

**Indirect Effect of X on Y through M**

```
         Effect     BootSE   BootLLCI   BootULCI
Ind1     -,0372      ,0409     -,1246      ,0410

Indirect Key
Ind1  'X'      ->       Mdiff    ->       Ydiff

********************** ANALYSIS NOTES AND WARNINGS ***********************

Bootstrap confidence interval method used: Percentile bootstrap.

Number of bootstrap samples for bootstrap confidence intervals:
  5000
```

```
The following variables were mean centered prior to analysis:
 (        PA_t2     +       PA_t1    )        /2

Level of confidence for all confidence intervals in output:
      95,00

------ END MATRIX -----
```

## References


Judd, C.M., Kenny, D.A., and McClelland, G.H. (2001). Estimating and testing mediation and moderation in within-subject designs. *Psychological methods* 6(2)**,** 115.

Montoya, A.K., and Hayes, A.F. (2017). Two-condition within-participant statistical mediation analysis: A path-analytic framework. *Psychological Methods* 22(1)**,** 6.
